# Mechanistic Studies on Trichoacorenol Synthase from *Amycolatopsis benzoatilytica*

**DOI:** 10.1002/cbic.201900584

**Published:** 2019-11-07

**Authors:** Jan Rinkel, Jeroen S. Dickschat

**Affiliations:** ^1^ Kekulé Institute of Organic Chemistry and Biochemistry University of Bonn Gerhard-Domagk-Strasse 1 53121 Bonn Germany

**Keywords:** enzyme mechanism, isotopic labeling, mass spectrometry, NMR spectroscopy, terpenes

## Abstract

Isotopic labeling experiments performed with a newly identified bacterial trichoacorenol synthase established a 1,5‐hydride shift occurring in the cyclization mechanism. During EI‐MS analysis, major fragments of the sesquiterpenoid were shown to arise via cryptic hydrogen movements. Therefore, the interpretation of earlier results regarding the cyclization mechanism obtained by feeding experiments in *Trichoderma* is revised.

## Graphical abstract

**Cryptic hydrogen movements**: A bacterial terpene synthase that produces the fungal sesquiterpene trichoacorenol was identified. Detailed labeling experiments revise a mechanistic pathway proposed for *Trichoderma*, and EI‐MS studies underpin the difficulties of conclusions from MS‐based feeding experiments in this system because of surprising hydrogen rearrangements.

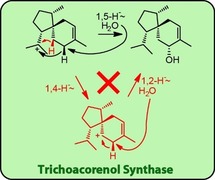

The cyclization mechanisms of terpene synthases (TSs) have fascinated natural product chemists for a long time and continue to do so, both for their complexity and for their biosynthetic relevance furnishing the largest group of natural products. TSs convert simple linear oligoprenyl diphosphates, such as farnesyl diphosphate (FPP), in a cationic cyclization cascade to form polycyclic hydrocarbons or alcohols with high stereocontrol.^1^ By providing a defined hydrophobic cavity in their active site, TSs promote a defined substrate folding and stabilization of transition states,^2^ thus circumventing the low selectivity of carbocationic reactions observed in solution. To differentiate between possible mechanistic pathways toward a terpenoid structure, isotopic labeling experiments constitute a reliable tool.^3^ In particular, feeding studies using labeled terpene precursors are a widely used approach to study TS mechanisms in their natural hosts. Also for the cyclization toward the fungal sesquiterpenoid trichoacorenol (**1**, Figure 1), this strategy was applied.^4^

**Figure 1:**
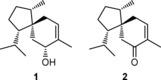
Structures of trichoacorenol (**1**) and acorenone (**2**).

Compound **1** was first isolated in 1968 from *Fusidium coccineum* as coccinol^5^ and later also found in *Trichoderma koningii*, where it was described with the currently used name trichoacorenol.^6^ In *Trichoderma* volatile profiles, **1** is often accompanied by its oxidation product acorenone (**2**).^7^ Besides syntheses yielding racemic compounds,^8^ synthetic approaches toward enantiomerically enriched **1** and **2** have also been described.^7^, ^9^ The cyclization mechanism is depicted in Scheme 1 and starts by isomerization of FPP to nerolidyl diphosphate (NPP), which is cyclized by diphosphate abstraction to the bisabolyl cation **A**, representing an important branching point in sesquiterpene biosynthesis.^10^ After a subsequent 1,2‐hydride shift to **B**, the spirocyclic center is formed by cyclization. Downstream of cation **C**, two pathways are discussed in the literature. The initially proposed pathway A involves a 1,5‐hydride shift followed by the attack of water to yield **1**.4a, 4b Based on feeding experiments of deuterated mevalonate isotopologues to *Trichoderma*, a second pathway B was invoked, which consists of a 1,4‐, 1,2‐hydride shift sequence.4c Herein we report labeling experiments using a recombinant TS for **1** from a bacterial source, using reliable NMR methods for the location of labels.

**Scheme 1:**
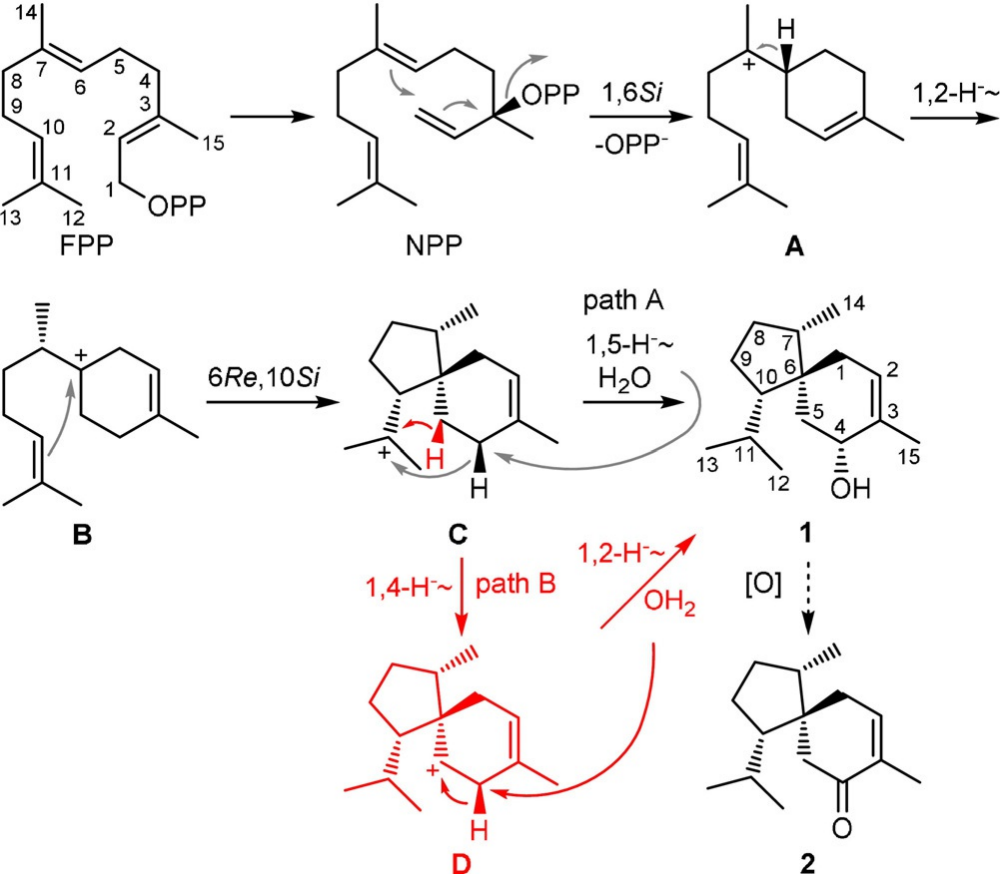
Cyclization of FPP to trichoacorenol (**1**) and oxidation to acorenone (**2**). Carbon numbering for **1** resembles the original positions in FPP.

As part of our ongoing search for novel bacterial TSs and interesting mechanisms, a TS (WP_020663213) from the clinical isolate *Amycolatopsis benzoatilytica* DSM 43387^11^ was cloned into the *Escherichia coli* expression vector pYE‐Express,^12^ which is phylogenetically unrelated to a characterized enzyme (Figure S1, Table S1 in the Supporting Information). The purified, recombinant protein (Figure S2) did not accept geranyl‐ (GPP), geranylgeranyl‐ (GGPP) or geranylfarnesyl diphosphate (GFPP), but converted FPP into a sesquiterpenoid alcohol **1**, which was detected by GC–MS (Figure 1) and identified by comparison with an authentic standard of synthetic *ent*‐**1** 7 as trichoacorenol (**1**). During GC–MS analysis of both samples, two acoradienes were observed, which are presumably formed by elimination of water from **1** during thermal impact in the GC inlet. Compounds **3** and **4** separated from *ent*‐**3** and *ent*‐**4** of synthetic origin on a homochiral GC column (Figure S3), thus establishing the absolute configuration of the enzyme product as (−)‐**1**, identical to previously described **1** from fungal sources. The same absolute configuration was independently concluded by comparing the NMR data of *ent*‐**1** (Table S2, Figures S4–S10) with HSQC spectra of labeled **1** obtained from enzyme incubations of the TS combined with FPP synthase^13^ from *Streptomyces coelicolor* and the selectively labeled samples (Table S3) (*R*)‐ and (*S*)‐(1‐^13^C,1‐^2^H)isopentenyl diphosphate (IPP)^14^ with IPP isomerase (IDI)^14^, ^15^ from *E. coli* (Figure S11) or (*Z*)‐ and (*E*)‐(4‐^13^C,4‐^2^H)IPP^16^ elongating dimethylallyl diphosphate (DMAPP; Figure S12). The TS from *A. benzoatilytica* is therefore characterized as a (−)‐(4*R*,6*S*,7*S*,10*S*)‐trichoacorenol (**1**) synthase (TaS).

Reliable peak assignment of the artificial elimination products **3** and **4** by their similar retention indices and mass spectra is difficult. Therefore, unambiguous identification was achieved by analysis of the products obtained from an incubation of TaS with (1,1‐^2^H_2_)FPP^17^ (Figure S13), in which two deuterium atoms were retained for **3**, but only one for **4**. The stereochemical courses for the elimination reactions were found to predominantly proceed through 1,2‐*syn* elimination for **3** and 1,4‐*syn* elimination for **4**, as followed by incubations of TaS with (*R*)‐ and (*S*)‐(1‐^2^H)GPP,^18^ IPP and FPPS, or (*R*)‐ and (*S*)‐(1‐^2^H)FPP,^17^ respectively (Figure S14). In cases of deuterium abstraction, a decreased stereoselectivity for these processes was observed.

Regarding the cyclization mechanism toward **1**, the 1,2‐hydride shift of the bisabolyl cation **A** toward **B** was followed by incubation of (3‐^13^C,2‐^2^H)GPP^19^ with IPP, FPPS and TaS, resulting in a triplet in the ^13^C NMR spectrum for C7 of **1** (Figure S15). To investigate the final hydride shift sequence toward **1** (path A or B, Scheme 1), the stereochemical identity of the shifting hydrogen atom from C4 was evident by ^13^C NMR analysis of the products obtained from (*Z*)‐ and (*E*)‐(4‐^13^C,4‐^2^H)IPP, DMAPP, FPPS and TaS. Whereas a triplet was observed for C4 in case of the *Z* sample, a singlet appeared in the *E* case (Figure 3 B, C), demonstrating a selective movement of H_*E*_. Its destination was targeted using (7‐^13^C)GPP^20^ and (*Z*)‐ or (*E*)‐(4‐^2^H)IPP^19^ with FPPS and TaS resulting in a complementary outcome (Figure 3 D, E). In conclusion, these results clearly show a 1,5‐hydride shift occurring in the cyclization toward **1** (path A). Whereas these findings are in line with the previously reported stereochemical course of the hydrogen shift from C4,4d they surprisingly contradict the described 1,4‐, 1,2‐hydride shift sequence (path B) deduced from feeding experiments in *Trichoderma*.4c Although two different mechanistic pathways operating in different TSs from *A. benzoatilytica* and *Trichoderma* converging to the same product cannot be ruled out, a unified pathway using the same intrinsic reactivity^21^ of cation **C** toward **1** seems more likely.

In previous studies, a proposed EI‐MS fragmentation mechanism of **1** (Scheme S1) was used to locate isotope incorporations in feeding experiments as a basis for conclusions on the terpene cyclization mechanism.4c, 4d Whereas the fate of the C4 hydrogens were deduced from the retro‐Diels–Alder (RDA) fragment *m*/*z* 84, the destination toward the isopropyl group was based on the two prominent diagnostic fragments *m*/*z* 138 and 151 (cf. Figure 2 B). Access to the TS for **1** gives the opportunity to reinvestigate its EI‐MS fragmentation using enzymatically prepared and NMR‐confirmed isotopologues of **1** from defined labeled terpene precursors. As expected, the product from the incubation visualized in Figure 3 D resulted in a +1 *m*/*z* shift in all three fragments by the incorporation of deuterium (Figure 4). Surprisingly, the mass spectrum from the corresponding experiment with deuterium being located at the isopropyl group (Figure 3 E) also showed an increase of *m*/*z* 138 and 151 by +1, whereas the RDA fragment exhibited no incorporation of label.

**Figure 2:**
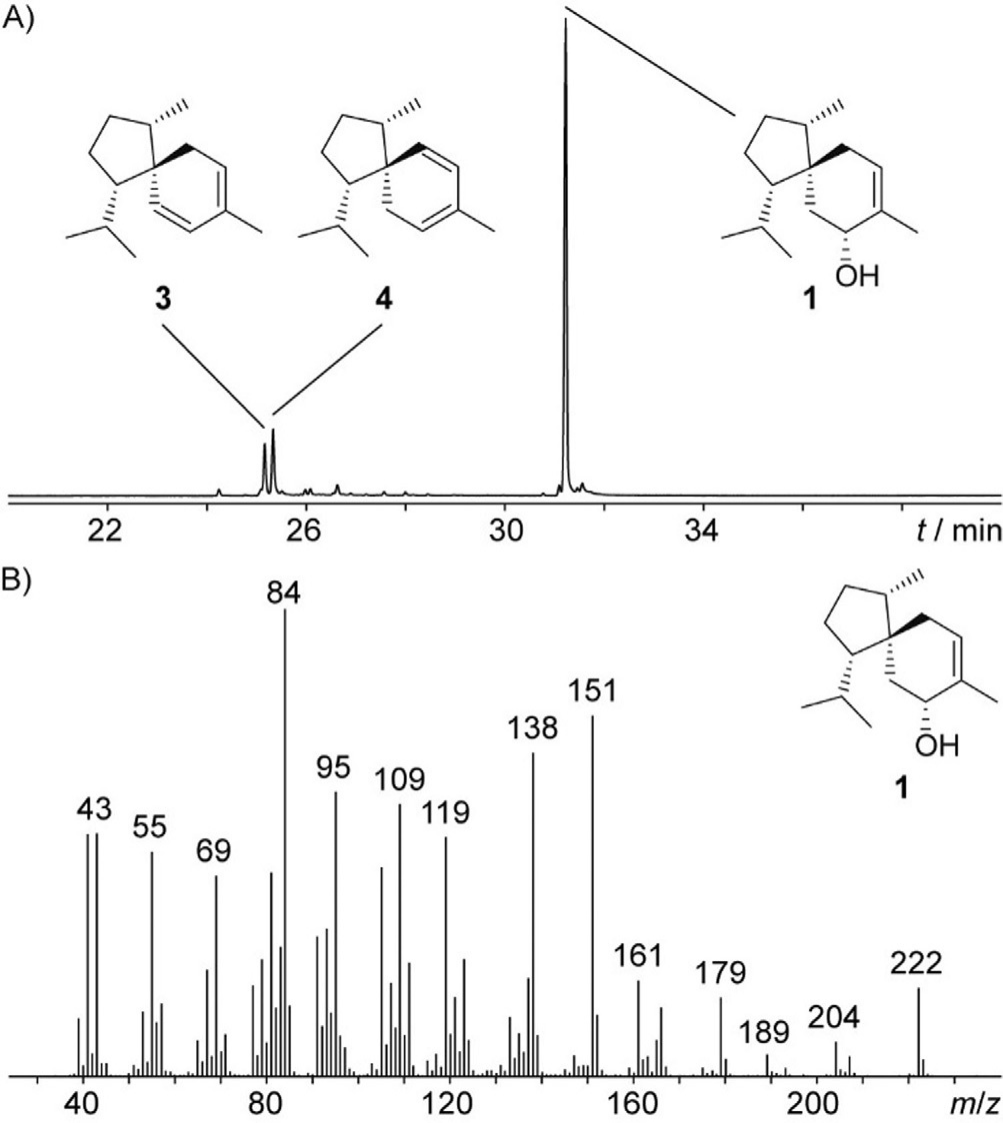
A) Total ion chromatogram of a hexane extract from the incubation of TaS with FPP and B) EI‐MS fragmentation spectrum of **1**.

**Figure 3:**
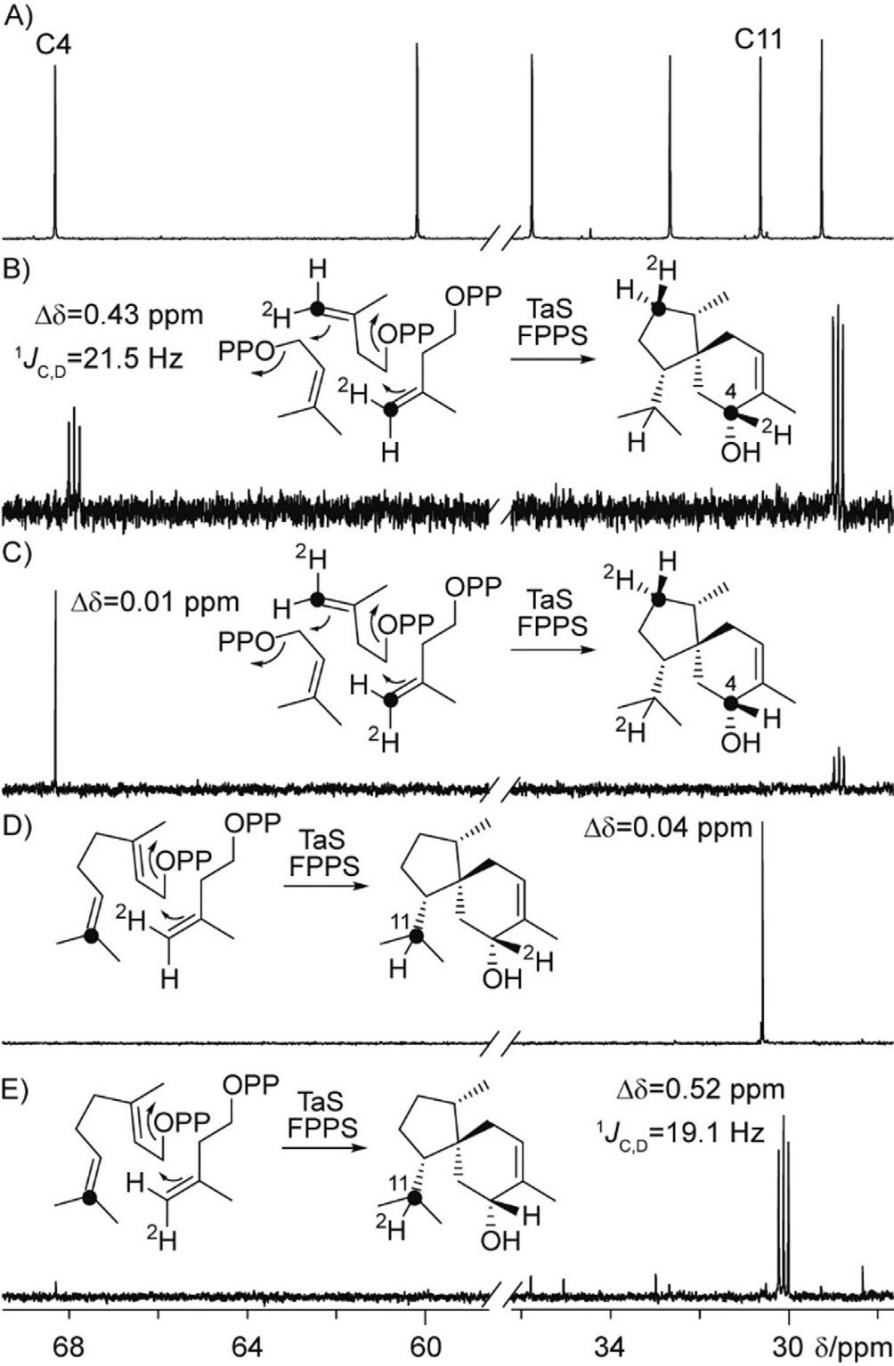
Partial ^13^C NMR spectra of A) unlabeled *ent*‐**1** (C_6_D_6_), and C_6_D_6_ extracts of incubation experiments with TaS and FPPS using B) (*Z*)‐(4‐^13^C,4‐^2^H)IPP+DMAPP, C) (*E*)‐(4‐^13^C,4‐^2^H)IPP+DMAPP, D) (7‐^13^C)GPP+(*Z*)‐(4‐^2^H)IPP, and E) (7‐^13^C)GPP+(*E*)‐(4‐^2^H)IPP demonstrating a selective movement of H_*E*_ from C4 to C11 (path A, Scheme 1). ^13^C‐Labeled carbons are represented by black dots.

**Figure 4:**
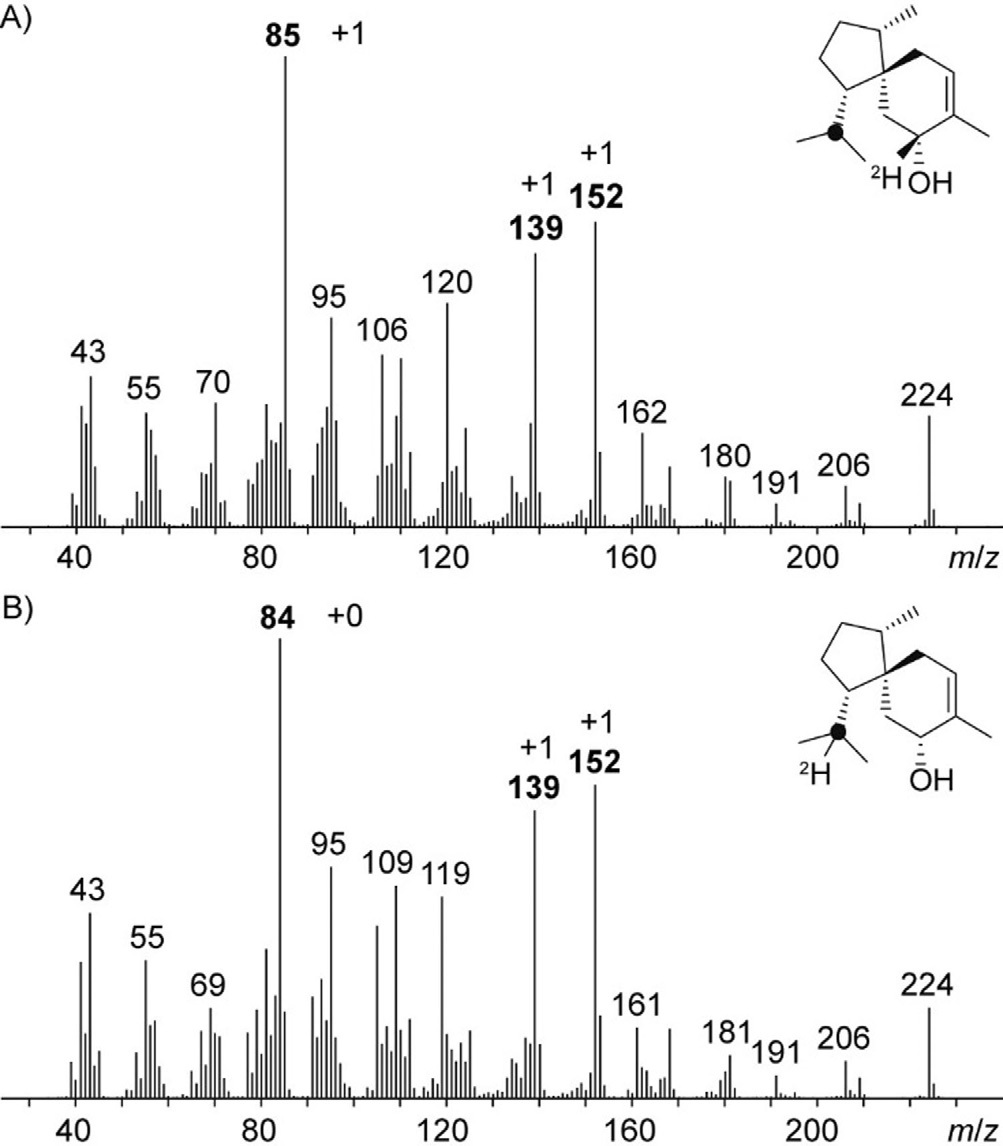
EI‐MS spectra of labeled **1** originating from incubations of TaS and FPPS with A) (7‐^13^C)GPP+(*Z*)‐(4‐^2^H)IPP (cf. Figure 3 D) and B) (7‐^13^C)GPP+(*E*)‐(4‐^2^H)IPP (cf. Figure 3 E). Diagnostic fragments with their mass shifts are shown in bold. Black dots represent ^13^C‐labeled carbon atoms.

These two spectra clearly demonstrate that *m*/*z* 138 and 151 cannot be used to locate the hydrogen atom involved in the 1,5‐hydride shift and therefore a differentiation of paths A and B based on the retainment of both deuterium atoms from C4 in these fragments is impossible. For a more systematic discussion of the EI‐fragmentation mechanism of **1**, all 15 ^13^C_1_ isotopomers of FPP^20^, ^22^ were converted by TaS, the selective incorporation of label was followed by ^13^C NMR (Figure S16) and EI‐MS spectra of each isotopomer of (^13^C_1_)‐**1** were recorded (Figure S17). Position‐specific mass shift analysis (PMA,22a, 23 Figure 5 A) confirmed the suggested carbon atoms resembling the three diagnostic fragments. Several incubation experiments with deuterated substrates were analyzed by GC–MS (Figure S18) revealing complex hydrogen movements for the fragment *m*/*z* 138, which are summarized in Figure 5 B (only the hydrogen atoms not resembling the PMA skeleton are shown). While H_a_ is part of the fragment (Figure 4), accompanied by a hydrogen atom from C9 (H_d_), hydrogen atoms from C5 (H_b_, high stereocontrol) and C1 (H_c_, low stereocontrol) are lost during fragmentation. One possible fragmentation mechanism explaining the experimentally observed hydrogen movements is shown in Scheme S2. However, for its complexity and for isotope effects promoting alternative fragmentations, other solutions may also apply.

**Figure 5:**
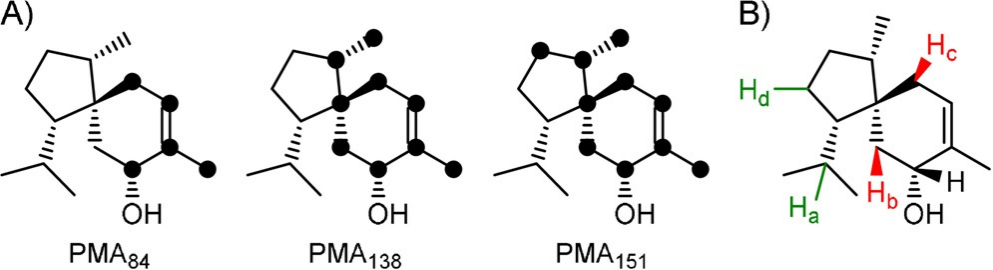
A) Position‐specific mass shift analysis (PMA) for diagnostic fragments of **1** (black dots represent carbon atoms contributing to a fragment) in agreement to previously proposed fragmentation mechanisms and B) results of hydrogen labeling experiments for *m*/*z* 138 revealing complex hydrogen fragmentations. Green hydrogens are part of this fragment whereas those colored in red are not.

In summary, a new bacterial sesquiterpene synthase from *A. benzoatilytica* was characterized as a trichoacorenol (**1**) synthase (TaS), representing the first characterized TS from this genus. Although **1** has been long known as a fungal metabolite, neither its occurrence in bacteria nor a producing enzyme from bacteria or fungi have been described. Because TaS does not have similarity to any fungal TS, horizontal gene transfer, as discussed recently for corvol ether synthase genes from bacteria^24^ and fungi,^25^ is unlikely. Instead, a convergent evolution as for fungal and bacterial phomopsene synthases^16^, ^26^ must be assumed. The cyclization mechanism of TaS was investigated in detail by isotopically labeled precursors and NMR analysis. Supporting the initial mechanistic suggestion,4a, 4b clear evidence for a 1,5‐hydride migration in the formation of **1** by bacterial TaS was obtained that is likely also relevant for the still unknown fungal trichoacorenol synthases. Additional EI‐MS studies suggested complex hydrogen movements to occur in the fragmentation mechanism toward diagnostic fragments of **1**. As demonstrated in this work, mechanistic conclusions from hydrogen positions determined by EI‐MS should be handled with care. Instead, NMR‐based methods provide solid access to the positions of labeled atoms within the target molecule, as discussed for the interesting case of trichoacorenol.

## Conflict of interest

*The authors declare no conflict of interest*.

## Supporting information

As a service to our authors and readers, this journal provides supporting information supplied by the authors. Such materials are peer reviewed and may be re‐organized for online delivery, but are not copy‐edited or typeset. Technical support issues arising from supporting information (other than missing files) should be addressed to the authors.

Supplementary
