# Supplementary material for: Mechanistic Studies on Trichoacorenol Synthase from Amycolatopsis benzoatilytica
Source: Chembiochem. 2019 Nov 7;21(6):807–10. doi: 10.1002/cbic.201900584 (PMC7155024; doi:10.1002/cbic.201900584)
Supplement: Supplementary file 1 — Supplementary [file CBIC-21-807-s001.pdf]

CHEMBIOCHEM

Supporting Information

Mechanistic Studies on Trichoacorenol Synthase from *Amycolatopsis benzoatilytica*

Jan Rinkel and Jeroen S. Dickschat*[a]

cbic_201900584_sm_miscellaneous_information.pdf

Accession: WP_020663213, locus-tag: AMYBE_RS0130650 (*Amycolatopsis benzoatilytica* DSM 43387), elongated by 42 amino acids assuming a different start codon

MVPEGEFPQQSLRIWPFDVPFSTRVNSERLRVSAHSRSWVQQMGLCTTAAELQRHDRYDMSLFACLNY
PDAAGADLDLISDWVCWWSVWNDLTDRPEFLHDPDRVTRFFSSLAAVVELSEREIDEEMPAQDNRFVV
AFSDIWRRWRRGMSAEFVSRTGRNWVNWFNSYIVECHNRHAGASLDVDTYHQIRDFTGAVILELDAAE
RVGHYEVPPELLEIPPVRAMREITVRVINITQDVQSLPKEEEAGDQHNLVLVLERRHSLTRMQALREV
HVMIRRWTDGFLAEEASVPRFLDQFEVPLARRRPVYKHIDNMRTLMKGGVEFCAASGRYVKL

A)

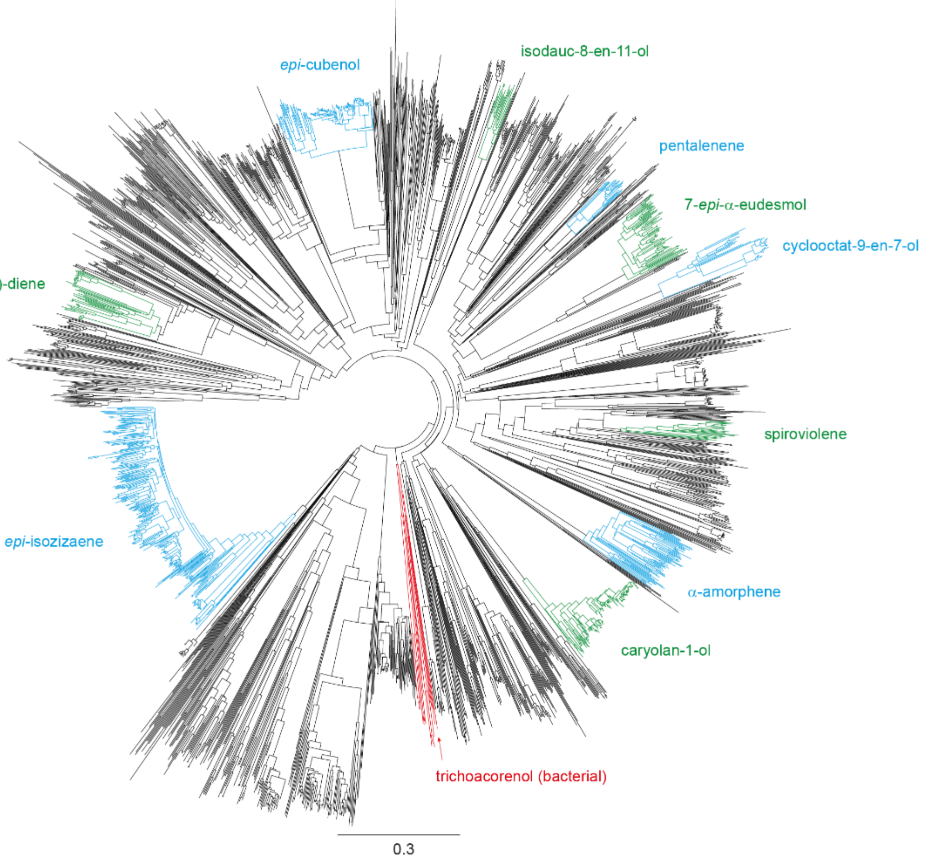

B)

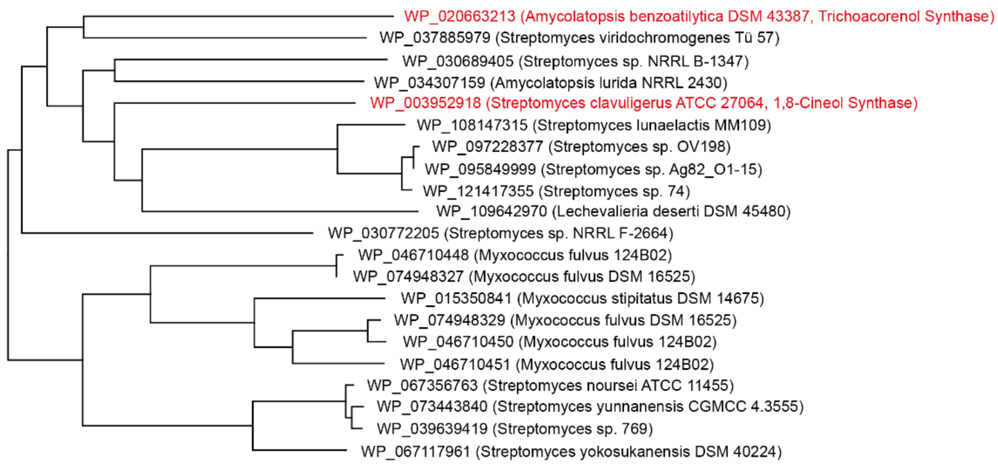

Figure S1. Amino acid sequence of trichoacorenol synthase (TaS, top). A different start codon for this gene was chosen to achieve a better alignment to known bacterial terpene synthases, therefore the shown sequence deviates by the green labelled amino acids from the NCBI database assignment. Highly conserved motifs are highlighted in yellow. A) Phylogenetic analysis of its relation towards 2400 bacterial terpene synthase sequences and B) detailed representation of the branch coloured in red. The closed characterised homologue is 1,8-cineol synthase (*Streptomyces clavuligerus*, 31% identity).

Strains, culture conditions and isolation of genomic DNA

Amycolatopsis benzoatilytica DSM 43387 was obtained from the Deutsche Sammlung von Mikroorganismen und Zellkulturen (DSMZ, Braunschweig, Germany). The cells were cultivated in GPHF-medium (100 mL; 10 g glucose, 5 g peptone from casein, 5 g yeast extract, 5 g beef extract, 740 mg $CaCl_2 \cdot 2\,H_2O$, 1 L water, pH 7.2) at 28 °C with shaking for 7 days. The bacterial cells were harvested by centrifugation (14600 g, 10 min) and resuspended in SET buffer (5 mL; 75 mM NaCl, 25 mM EDTA, 20 mM Tris/HCl, pH 8.0). Lysis was done by addition of lysozyme solution (100 μL; 50 mg/mL) followed by shaking for 30 min at 37 °C. Proteinase K solution (100 μL; 50 mg/mL) and SDS solution (600 μL; 10%) were added and the mixture was incubated for 1 h at 55 °C with shaking. Phenol/chloroform/isoamylalcohol (5 mL; 25:24:1) was added and the solution was mixed by inversion before centrifugation for 30 min at 14600 g. The aqueous layer was transferred to a fresh tube and ethanol (60% vol.) was added for precipitation of DNA. After centrifugation and washing with 70% ethanol the DNA was redissolved in water to a concentration of ca. 1000 ng/μL.[1]

Table S1. Primers used for gene cloning.

Primer	Sequence[a]
JR145f_WP020663213	ATGGTTCCCGAAGGAGAATTTCCTCAGC
JR145r_WP020663213	TCAGAGTTTCACGTAGCGCCCG
JR146f_WP020663213	GGCAGCCATATGGCTAGCATGACTGGTGGAATGGTTCCCGAAGGAGAATTTCCTCAGC
JR146r_WP020663213	TCTCAGTGGTGGTGGTGGTGGTGCTCGAGTTCAGAGTTTCACGTAGCGCCCG

[a] Homology arms for recombination in yeast matching the terminal sequences of linearised pYE-Express (HindIII and EcoRI digestion) are underlined.

Gene cloning

The target gene was amplified from gDNA by PCR using Q5 high-fidelity DNA polymerase (New England Biolabs, Ipswich, MA, USA) and the short primer pairs listed in Table S1. PCR conditions were: initial denaturation at 98 °C for 30 s; cyclic programme with melting at 98 °C for 10 sec, annealing at 60 – 70 °C for 30 sec, elongation at 72 °C for 30 sec, repeated 33 times; final elongation at 72 °C for 5 min. The obtained PCR product was elongated by homology arms matching the terminal sequences of the linearised expression vector pYE-Express (HindIII and EcoRI digestion) via a second PCR using the corresponding long primer pair in Table S1. Yeast homologous recombination of the elongated PCR product with linearised pYE-Express[2] was carried out through the standard protocol using LiOAc, polyethylene glycol and salmon sperm DNA.[3] After yeast transformation cultures were grown on SM-URA agar (425 mg yeast nitrogen base, 1.25 g ammonium sulphate, 5 g glucose, 192.5 mg nutritional supplement minus uracil, 5 g agar, 250 mL water) at 28 °C for 3 days. The recombinant plasmid was isolated from grown yeast colonies using the Zymoprep Yeast Plasmid Miniprep II kit (Zymo Research, Irvine, CA, USA) and subsequently used for transformation of *E. coli* BL21(DE3) electrocompetent cells. Cells were plated on LB agar plates with kanamycin (50 μg/mL) followed by incubation at 37 °C overnight. Single colonies were selected and used to inoculate LB medium (6 mL) liquid cultures with kanamycin (6 μL; 50 mg mL^{-1}). After 8 h growth at 37 °C, plasmid DNA was isolated and checked for correct insertion of the desired gene by analytical digest with XhoI and PvuII and by sequencing.

Gene expression and protein purification

A preculture of *E. coli* BL21(DE3) transformed with pYE-WP020663213 (TaS) was grown in LB medium with kanamycin (50 μg/mL) overnight with shaking at 37 °C. Main cultures were inoculated with the preculture (1/1000) and grown in LB medium containing kanamycin (50 μg/mL) with shaking at 37 °C until OD_{600} = 0.4 – 0.6 was reached. Expression was induced by the addition of aqueous IPTG solution (400 mM, 1/1000) after cooling the cultures to 18 °C. The cultures were shaken at 18 °C overnight. The cells were harvested via centrifugation (9600 g, 3 min, 4 °C), resuspended in binding buffer (10 mL L^{-1} culture; 20 mM Na_2HPO_4, 500 mM NaCl, 20 mM imidazole, 1 mM $MgCl_2$, pH = 7.4, 4 °C) and lysed by ultra-sonication (5x 30 s, 50% power) on ice. The cell debris was removed by centrifugation (14600 g, 7 min, 4 °C) and the soluble protein fraction was loaded onto Ni^{2+}-NTA superflow affinity chromatography columns (Qiagen, Venlo, Netherlands) equilibrated with binding buffer. The columns were washed with binding buffer (2x 10 mL L^{-1} culture) and the desired proteins were eluted with elution buffer (2x 6.25mL L^{-1} culture; 20 mM Na_2HPO_4, 500 mM NaCl, 500 mM imidazole, 1 mM $MgCl_2$, pH = 7.4, 4 °C). The protein concentration was determined by Bradford assay.[4] A typical protein concentration by this procedure was 2.4 mg/mL for TaS.

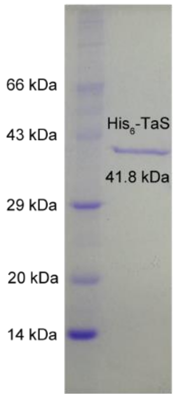

Figure S2. SDS-PAGE analysis of recombinant TaS (calculated molecular weight: 41.8 kDa) from *A. benzoatilytica*.

GC/MS analyses

GC/MS analyses were performed on a 7890B GC – 5977A mass detector system (Agilent, Santa Clara, CA, USA). A HP5-MS fused silica capillary column (30 m, 0.25 mm i. d., 0.50 μm film) was used in the GC. GC parameters were 1) inlet pressure: 77.1 kPa, He at 23.3 mL min^{-1}, 2) injection volume: 2 μL, 3) temperature program: 5 min at 50 °C increasing at 5 °C min^{-1} to 320 °C, 4) 60 s valve time, and 5) carrier gas: He at 1.2 mL min^{-1}. MS parameters were 1) source: 230 °C, 2) transfer line: 250 °C, 3) quadrupole: 150 °C and 4) electron energy: 70 eV. Retention indices (*I*) were determined in comparison to a homologous series of *n*-alkanes (C_7-C_{40}).

NMR spectroscopy

NMR spectra were recorded on a Bruker (Billerica, MA, USA) Avance III HD Prodigy (500 MHz) or an Avance III HD Cryo (700 MHz) NMR spectrometer. Spectra were referenced against solvent signals (^{1}H-NMR, residual proton signals: C_6D_6 δ = 7.16, ^{13}C-NMR: C_6D_6 δ = 128.06).[5]

Incubation experiments with recombinant TaS

The diphosphates GPP, FPP, GGPP or GFPP (1 mg) were dissolved in substrate buffer (1 mL; 25 mM NH_4HCO_3) and diluted with binding buffer (3.5 mL) and incubation buffer (5 mL; 50 mM Tris/HCl, 10 mM $MgCl_2$, 20% glycerol, pH = 8.2). Elution fraction of TaS (0.5 mL) was added to each sample followed by incubation with shaking at 28 °C for 4 h. The products were extracted with hexane (100 μL), the extracts were dried with $MgSO_4$ and analysed by GC/MS. Only for FPP, formation of a product was observed (Figure 2).

Chiral GC/MS analysis

The GC/MS system described above was equipped with an Agilent Cyclosil-B capillary column (30 m, 0.25 mm inner diameter, 0.25 µm film) to record GC/MS data on a homochiral stationary phase. For analysis of acoradienes **3** and **4**, the GC was programmed as follows: starting from 100 °C, increasing with 1 °C/min to 160 °C, then further increasing with 40 °C/min to 245 °C while holding this temperature for 1 min.

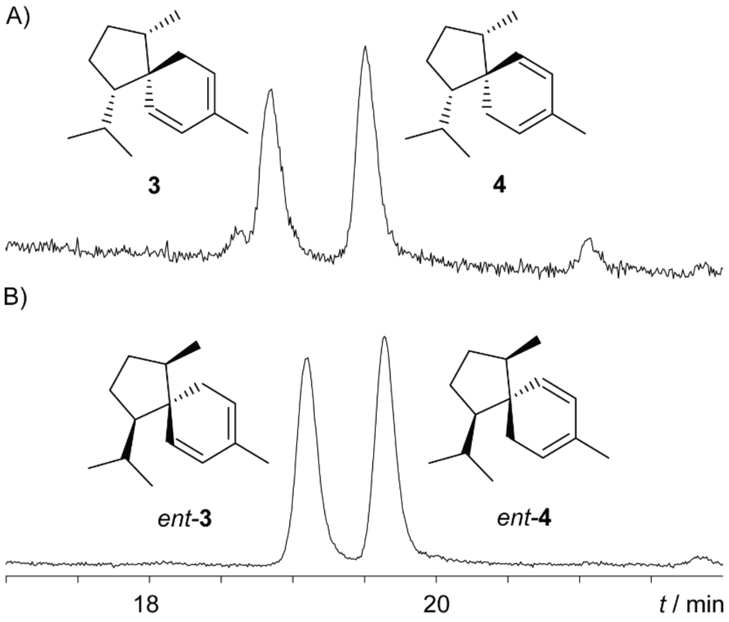

Figure S3. Total ion chromatograms on a homochiral stationary phase of A) acoradienes **3** and **4** originating from an FPP incubation with TaS and B) *ent*-**3** and *ent*-**4** formed during the analysis of synthetic *ent*-**1**.[6]

Table S2. NMR data of trichoacorenol (*ent*-**1**)[6] in C_6D_6 recorded at 298 K.

C[a]		^1H[b]	^{13}C[b]
1	CH_2	2.03 (m, H_α)	35.8
		1.68 (m, H_β)	
2	CH	5.37 (m)	124.5
3	C_q	–	136.8
4	CH	4.14 (m)	68.3
5	CH_2	1.59 (m, H_β)	32.7
		1.21 (dd, ^2J = 13.3, ^3J = 9.8, H_α)	
6	C_q	–	45.2
7	CH	1.44 (m)	47.0
8	CH_2	1.63 (m, H_β)	29.3
		1.11 (m, H_α)	
9	CH_2	1.65 (m, H_β)	27.0
		1.31 (m, H_α)	
10	CH	1.10 (m)	60.2
11	CH	–	30.6
12	CH_3	0.93 (d, ^3J = 6.5, 3H)	23.5
13	CH_3	0.83 (d, ^3J = 6.5, 3H)	23.3
14	CH_3	0.81 (d, ^3J = 6.8, 3H)	14.5
15	CH_3	1.81 (m, 3H)	19.4

[a] Carbon numbering corresponding to FPP as shown in Scheme 1 of main text. [b] Chemical shifts δ in ppm, multiplicity: s = singlet, d = doublet, m = multiplet, coupling constants J are given in Hertz.

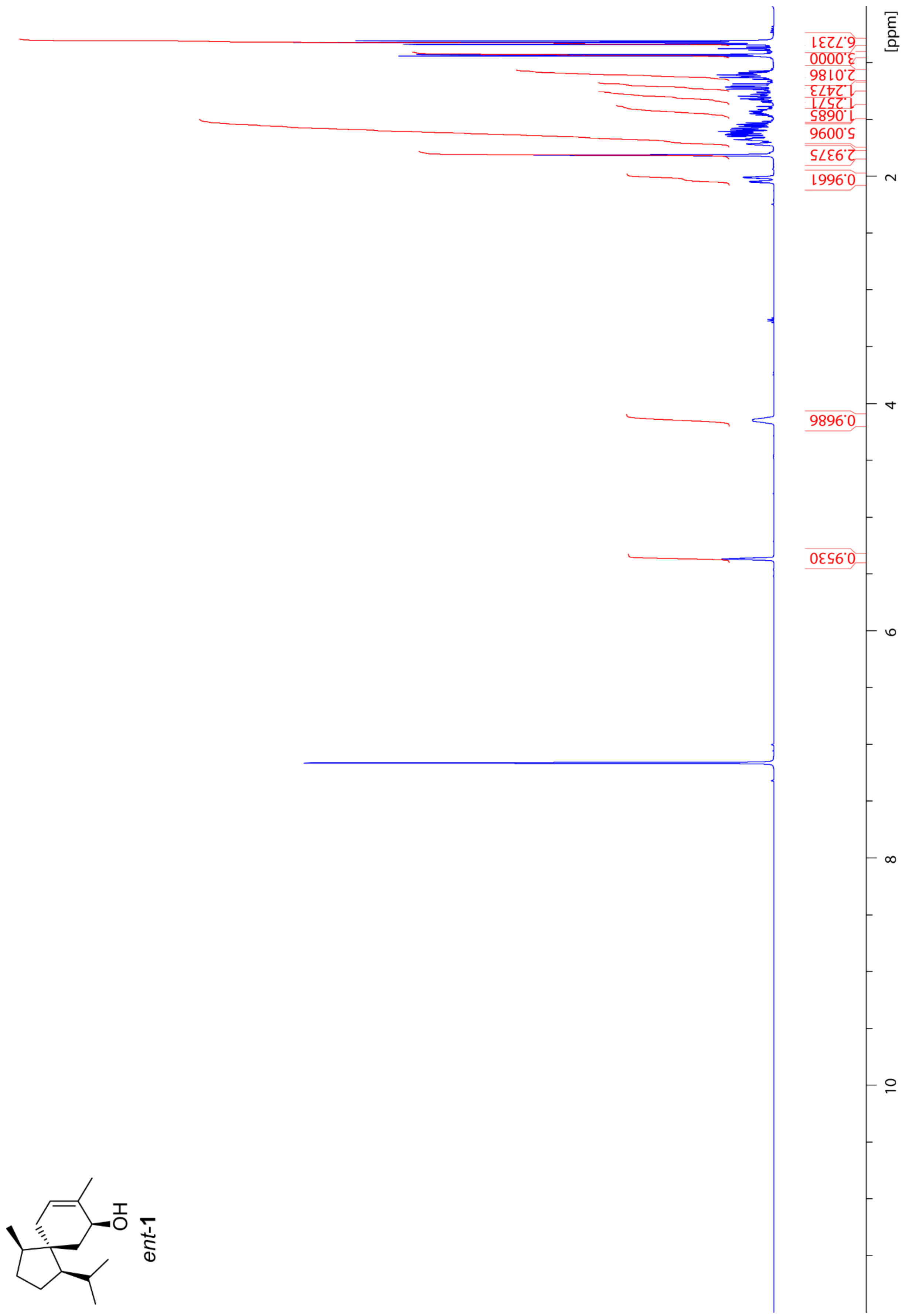

Figure S4. ^{1}H NMR spectrum of *ent*-**1** (500 MHz, C_6D_6).

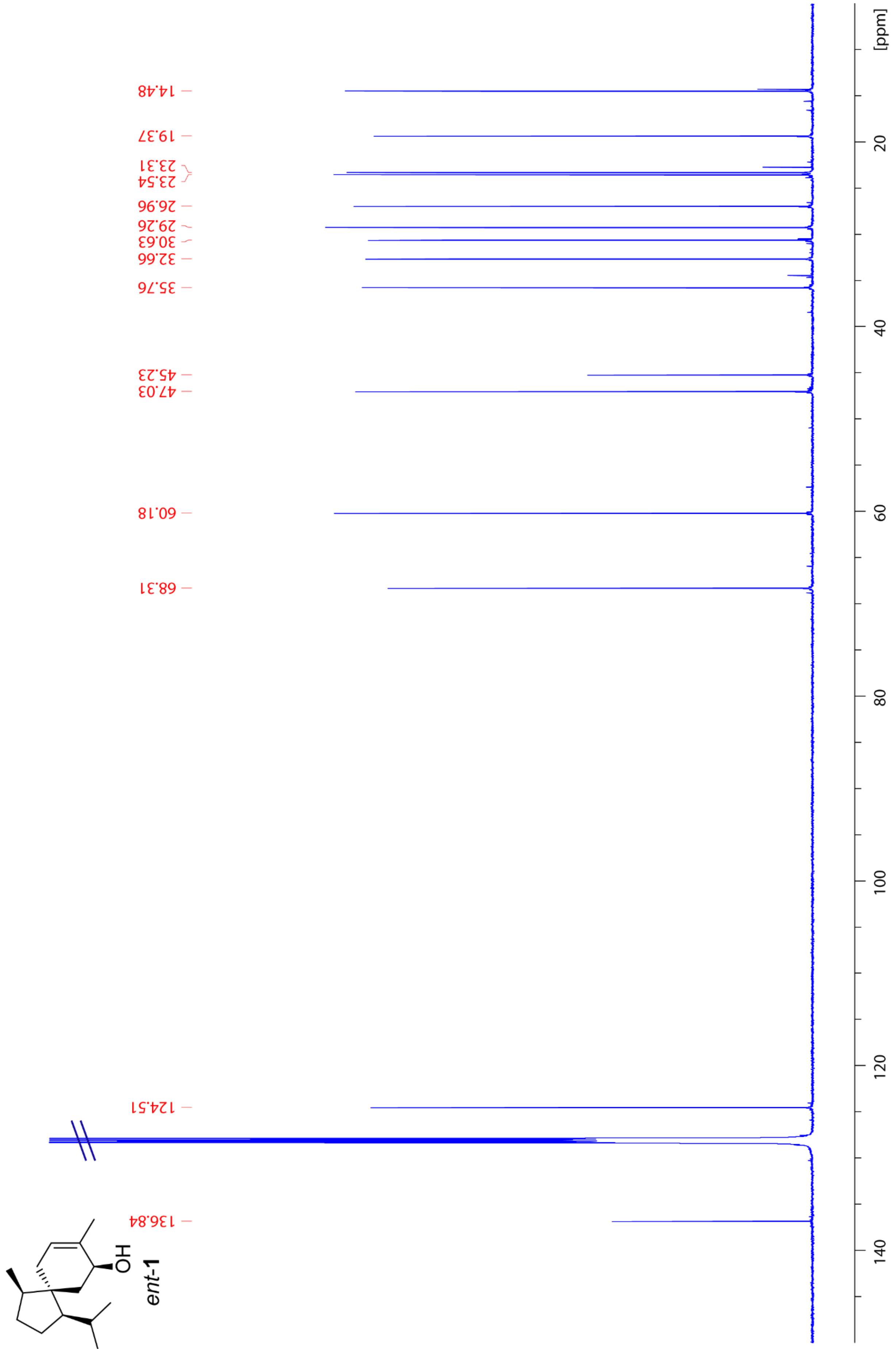

Figure S5. ^{13}C NMR spectrum of *ent*-**1** (126 MHz, C_6D_6).

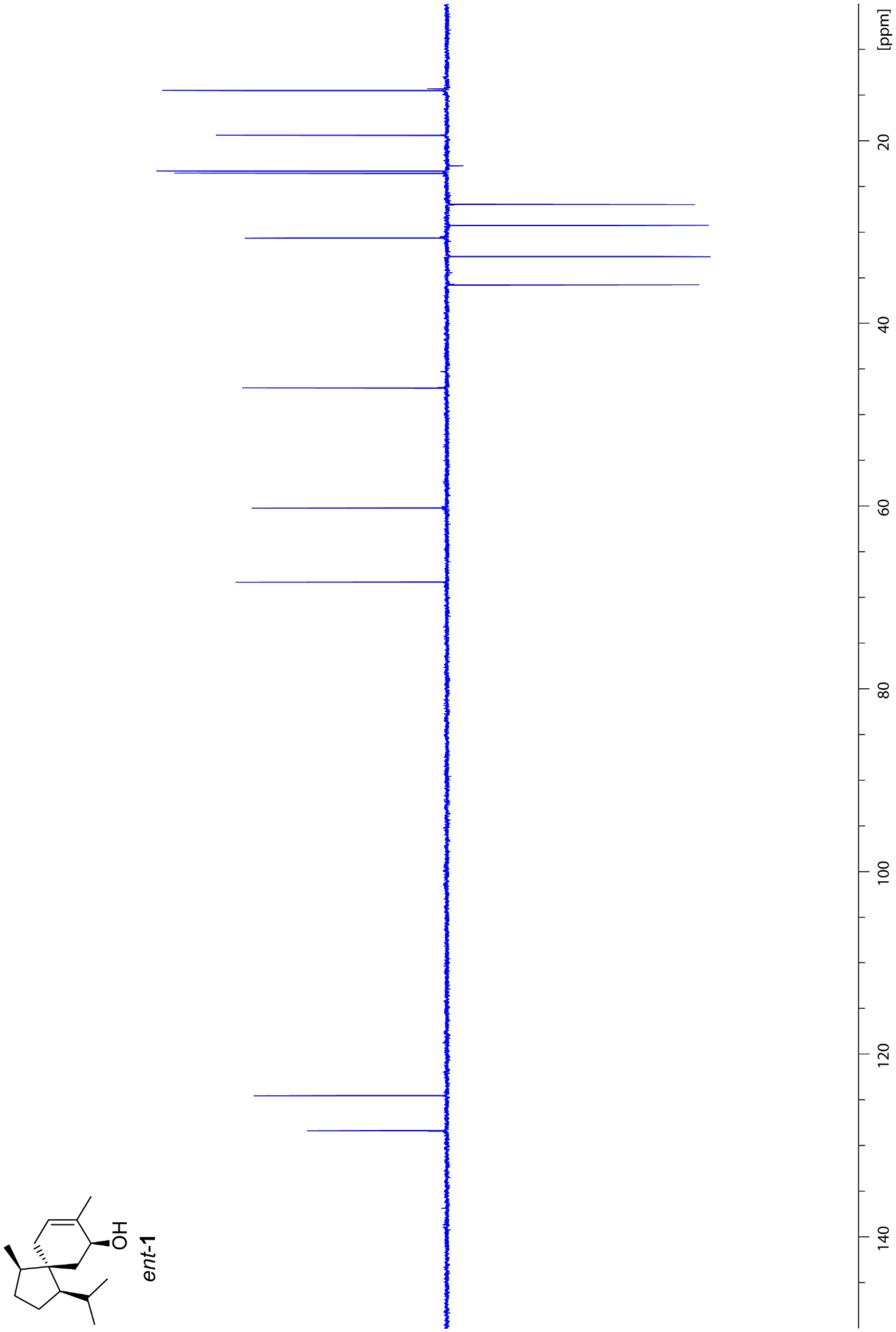

Figure S6. ^{13}C-DEPT-135 spectrum of *ent*-**1** (126 MHz, C_6D_6).

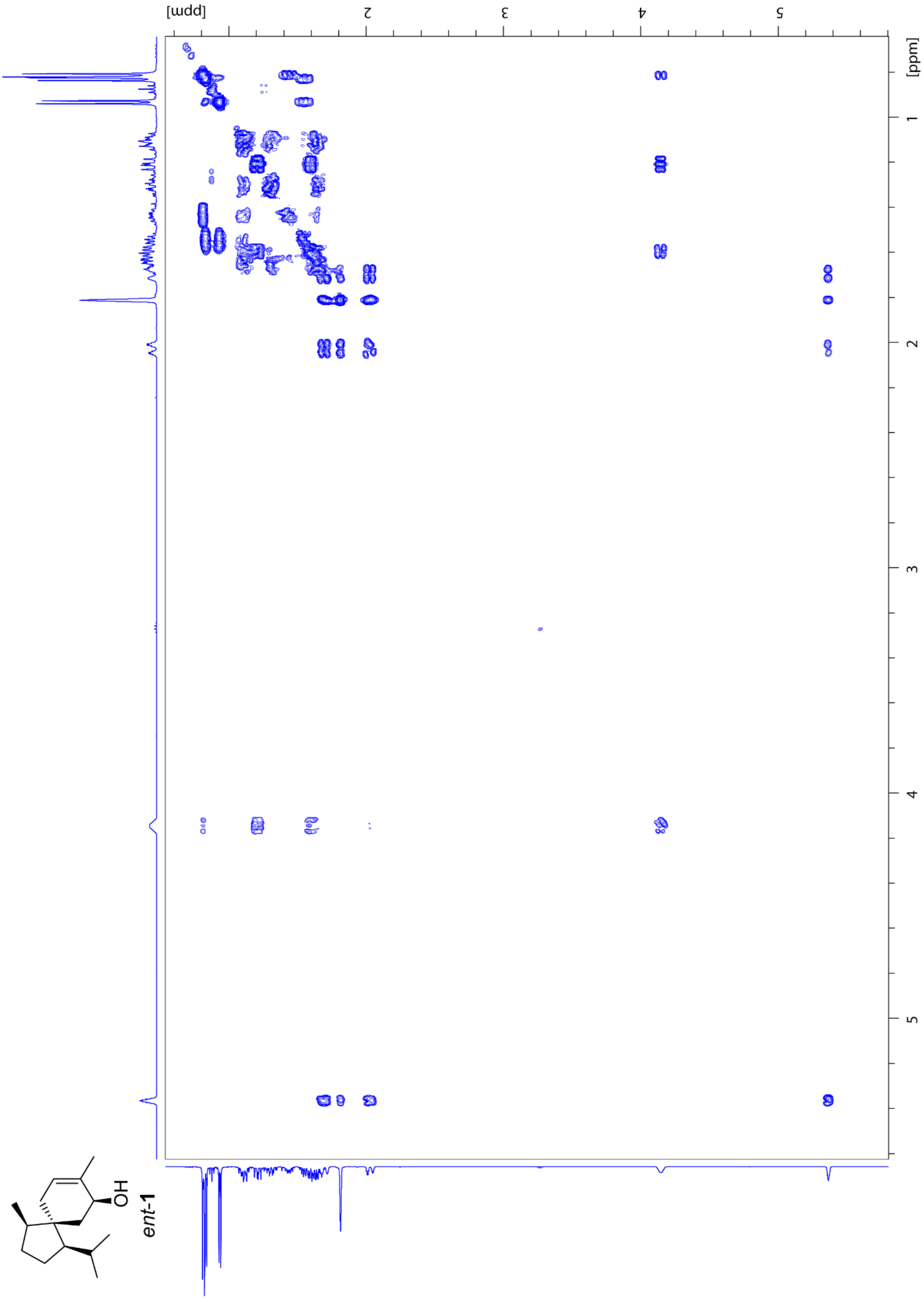

Figure S7. $^{1}H,^{1}H$-COSY spectrum of *ent*-**1** (500 MHz, C_6D_6).

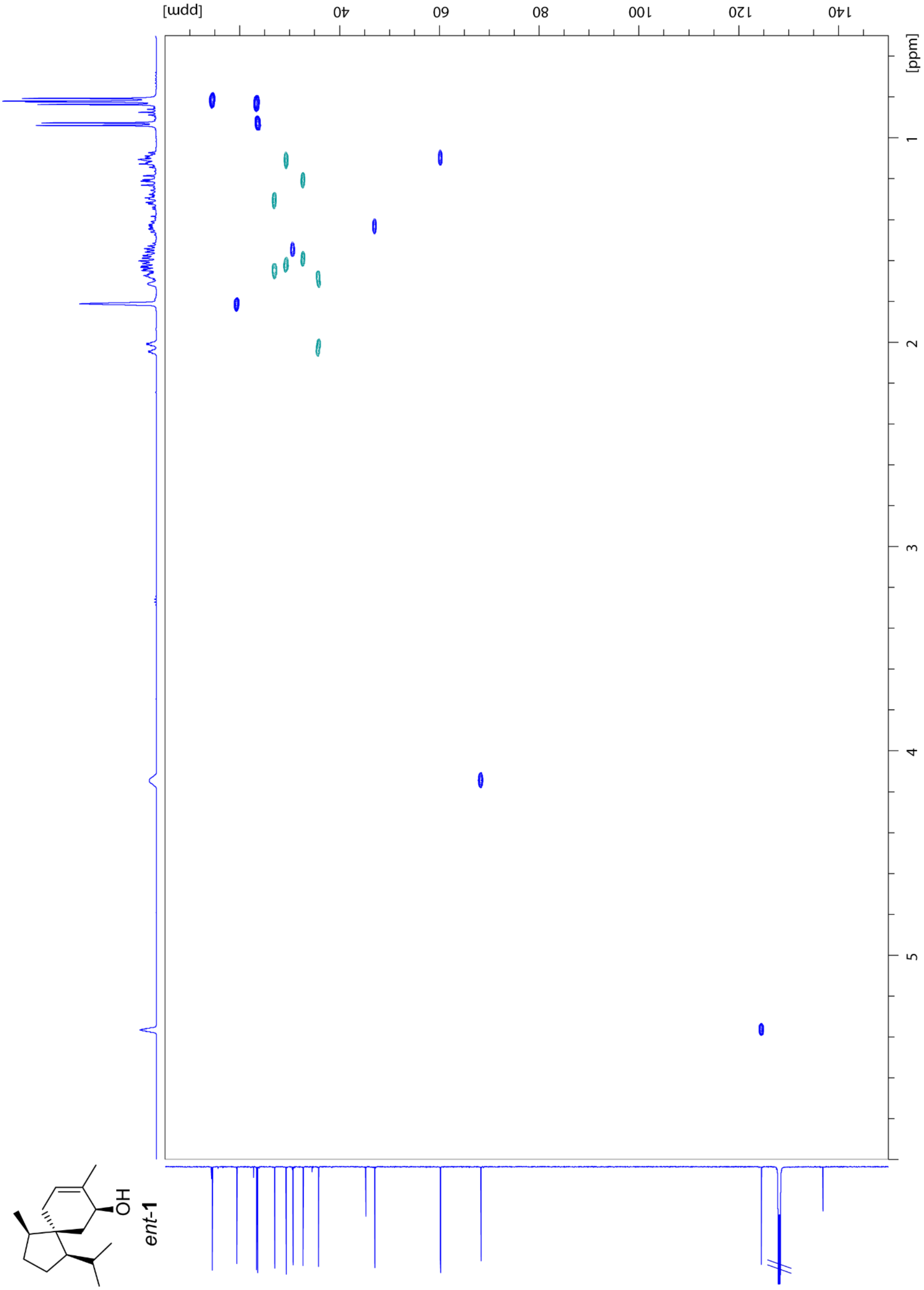

Figure S8. HSQC spectrum of *ent*-**1** (500 MHz, C_6D_6).

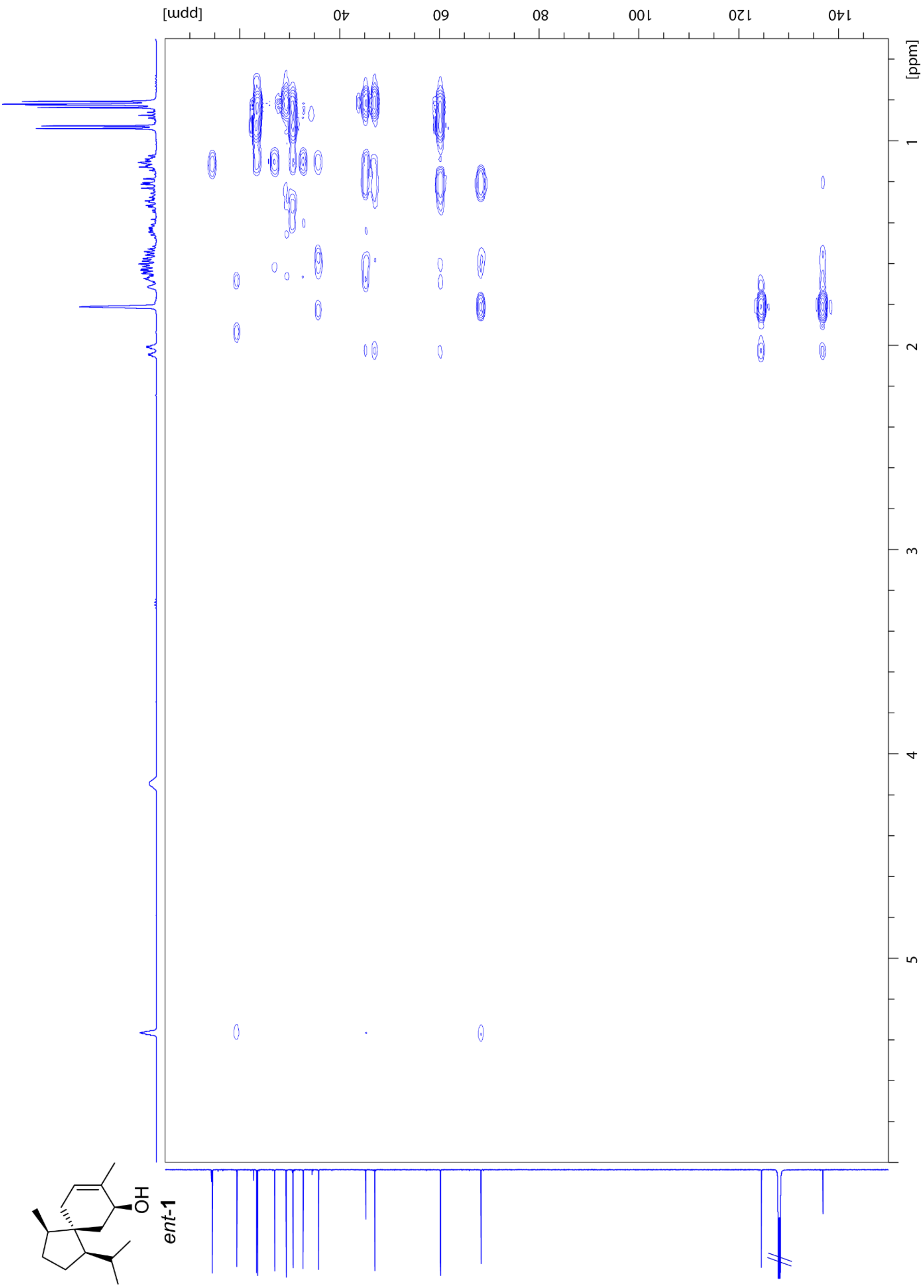

Figure S9. HMBC spectrum of *ent*-**1** (500 MHz, C_6D_6).

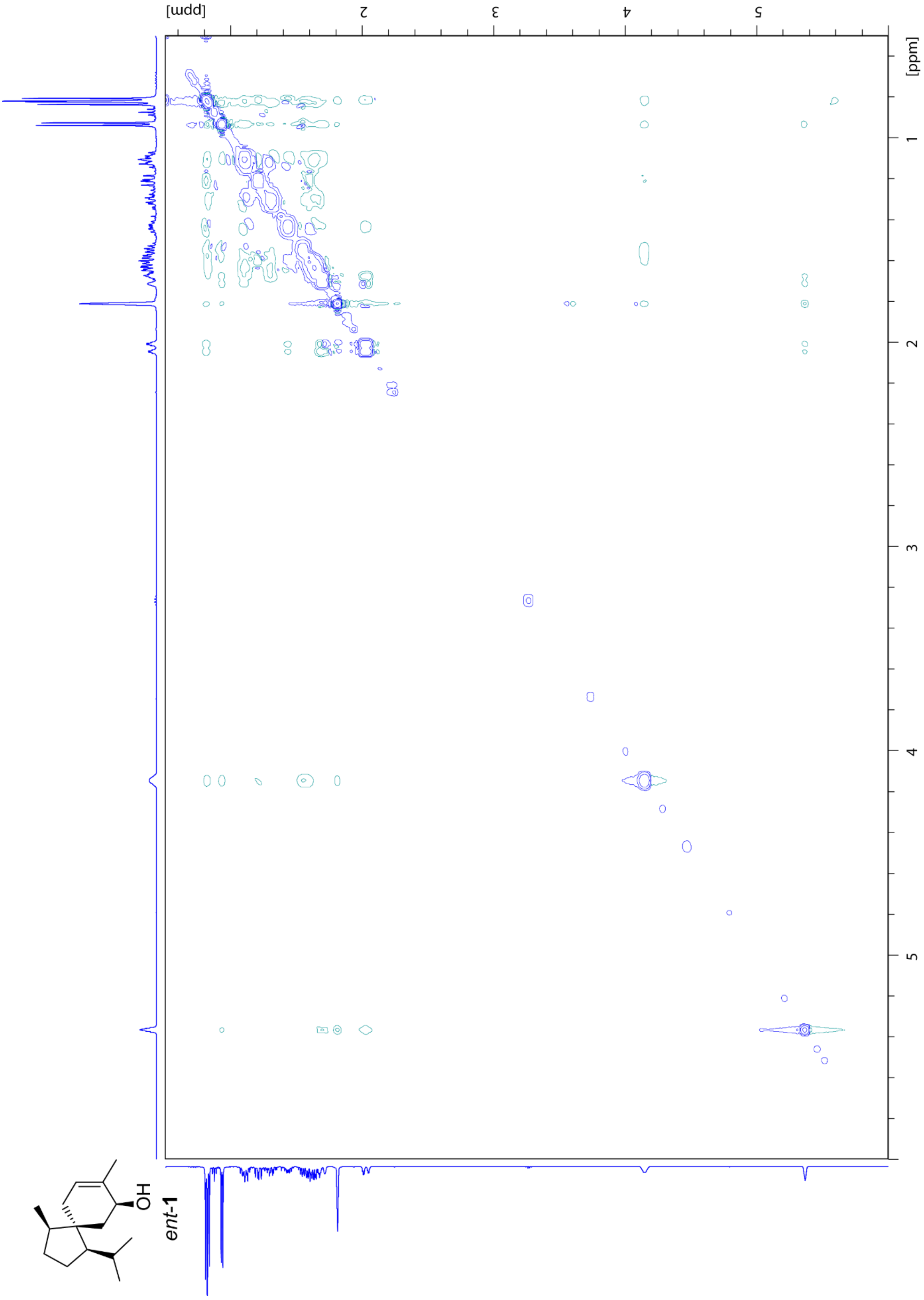

Figure S10. NOESY spectrum of *ent*-**1** (500 MHz, C_6D_6).

Incubation experiments with isotopically labelled substrates and TaS

Isotopic labelling experiments were performed with amounts of ca. 1 mg labelled FPP (or its precursors) in substrate buffer (1 mL), incubation buffer (5 mL), enzyme elution fractions and binding buffer (to 10 mL total volume) with the substrates and enzyme preparations as listed in Table S3. After incubation with shaking at 28 °C for 4 h, the products were extracted with C_6D_6 twice (650 μL and 300 μL) and the extracts were analysed by NMR and/or GC/MS.

Table S3. Isotopic labelling experiments with TaS.

entry	substrate(s)	enzyme(s)	results shown in
1	(*R*)-(1-^{13}C,1-^{2}H)IPP[7]	TaS, IDI,[7] FPPS[8]	Figure S11
2	(*S*)-(1-^{13}C,1-^{2}H)IPP[7]	TaS, IDI, FPPS	Figure S11
3	DMAPP + (*Z*)-(4-^{13}C,4-^{2}H)IPP[9]	TaS, FPPS	Figures 3 and S12
4	DMAPP + (*E*)-(4-^{13}C,4-^{2}H)IPP[9]	TaS, FPPS	Figures 3 and S12
5	(1,1-$^{2}H_2$)FPP[10]	TaS	Figures S13 and S18
6	(*R*)-(1-^{2}H)GPP[11] + IPP	TaS, FPPS	Figures S14 and S18
7	(*S*)-(1-^{2}H)GPP[11] + IPP	TaS, FPPS	Figures S14 and S18
8	(*R*)-(1-^{2}H)FPP[10]	TaS	Figures S14 and S18
9	(*S*)-(1-^{2}H)FPP[10]	TaS	Figures S14 and S18
10	(3-^{13}C,2-^{2}H)GPP[12] + IPP	TaS, FPPS	Figures S15 and S18
11	(7-^{13}C)GPP[13] + (*Z*)-(4-^{2}H)IPP[12]	TaS, FPPS	Figures 3 and 4
12	(7-^{13}C)GPP + (*E*)-(4-^{2}H)IPP[12]	TaS, FPPS	Figures 3 and 4
13	(1-^{13}C)FPP[14]	TaS	Figures S16 and S17
14	(2-^{13}C)FPP[14]	TaS	Figures S16 and S17
15	(3-^{13}C)FPP[14]	TaS	Figures S16 and S17
16	(4-^{13}C)FPP[14]	TaS	Figures S16 and S17
17	(5-^{13}C)FPP[14]	TaS	Figures S16 and S17
18	(6-^{13}C)FPP[14]	TaS	Figures S16 and S17
19	(7-^{13}C)FPP[14]	TaS	Figures S16 and S17
20	(8-^{13}C)FPP[14]	TaS	Figures S16 and S17
21	(9-^{13}C)FPP[14]	TaS	Figures S16 and S17
22	(2-^{13}C)DMAPP[15] + IPP	TaS, FPPS	Figures S16 and S17
23	(11-^{13}C)FPP[14]	TaS	Figures S16 and S17
24	(12-^{13}C)FPP[14]	TaS	Figures S16 and S17
25	(9-^{13}C)GPP[16] + IPP	TaS, FPPS	Figures S14 and S78
26	(10-^{13}C)GPP[13] + IPP	TaS, FPPS	Figures S16 and S17
27	(15-^{13}C)FPP[14]	TaS	Figures S16 and S17
28	(2-^{13}C,1,1-$^{2}H_2$)DMAPP[8] + IPP	TaS, FPPS	Figure S18
29	(2-^{2}H)DMAPP[17] + IPP	TaS, FPPS	Figure S18
30	DMAPP + (4,4-$^{2}H_2$)IPP[13]	TaS, FPPS	Figure S18

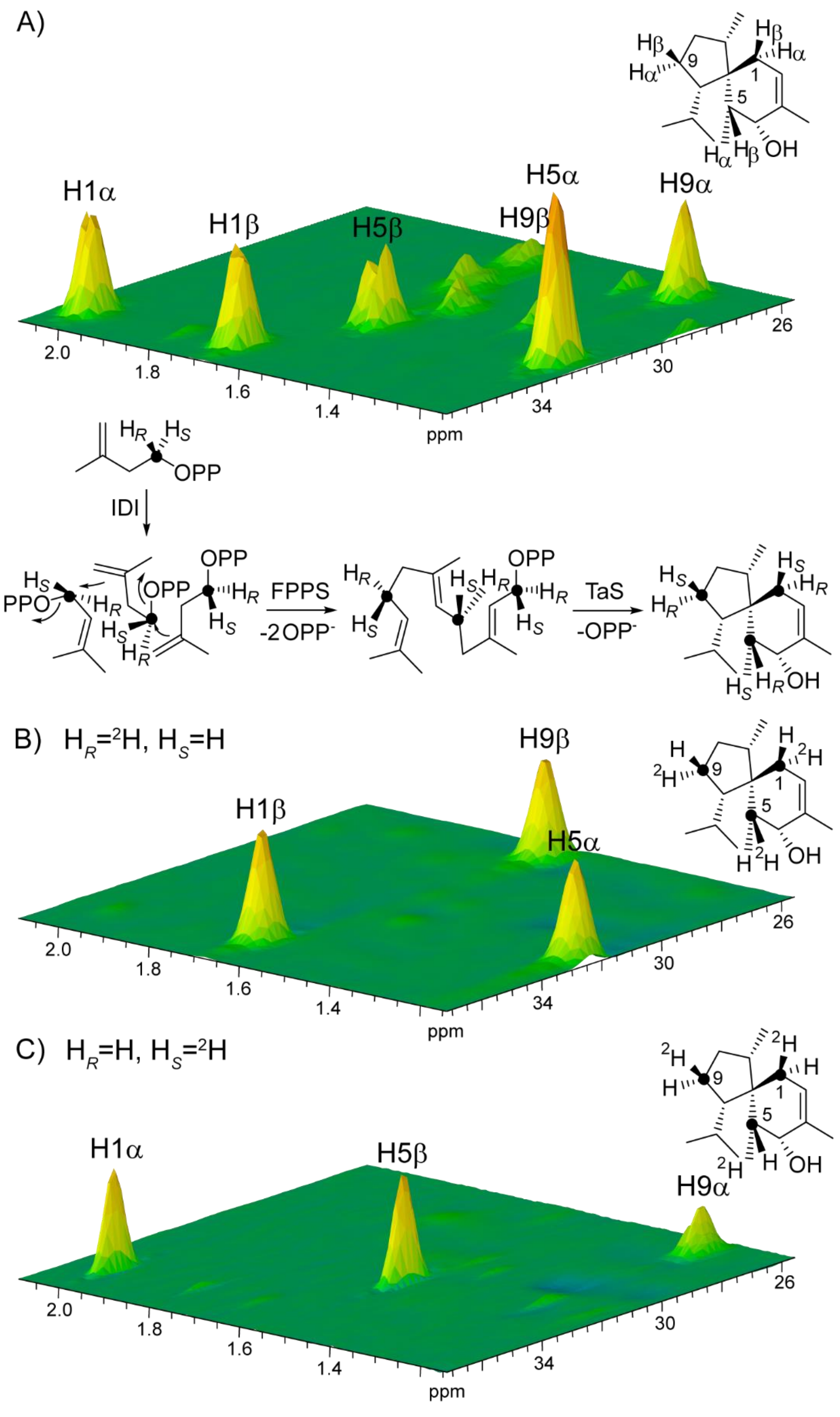

Figure S11. Determination of the absolute configuration of **1** using the substrates (*R*)- and (*S*)-(1-^{13}C,1-^{2}H)IPP. A) HSQC spectrum of unlabelled *ent*-**1**. B) HSQC spectrum of labelled **1** obtained from (*R*)-(1-^{13}C,1-^{2}H)IPP. C) HSQC spectrum of labelled **1** obtained from (*R*)-(1-^{13}C,1-^{2}H)IPP. The selective incorporation of deuterium can be used to determine the absolute configuration at the investigated methylene positions C1, C5 and C9. Combining these information with the relative configuration obtained by NOESY results in the absolute configuration for **1** as shown. Black dots represent ^{13}C-labelled carbon atoms.

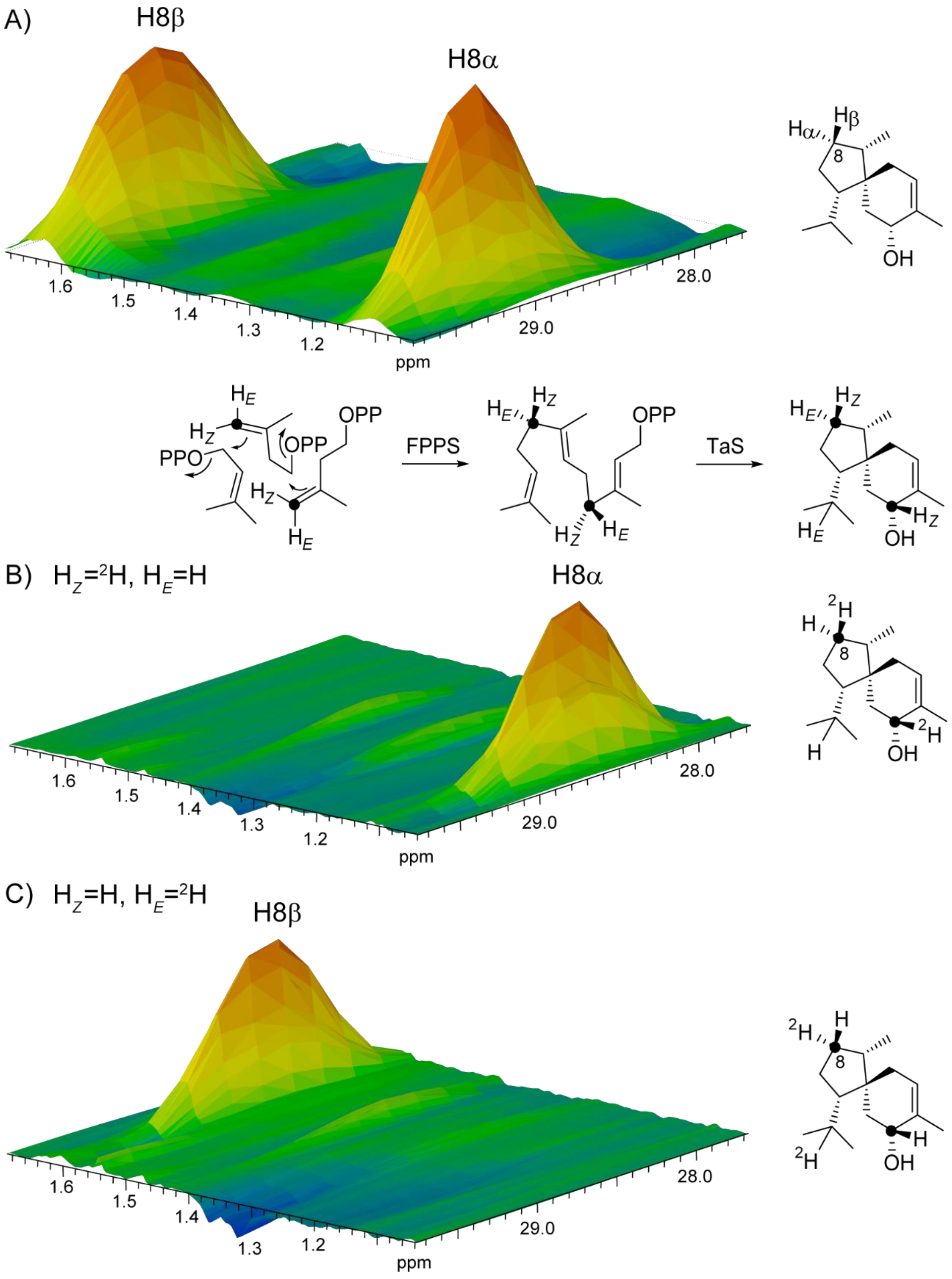

Figure S12. Determination of the absolute configuration of **1** using the substrates (*Z*)- and (*E*)-(4-^{13}C,4-^{2}H)IPP. A) Partial HSQC spectrum of unlabelled *ent*-**1**. B) Partial HSQC spectrum of labelled **1** obtained from (*Z*)-(4-^{13}C,4-^{2}H)IPP. C) Partial HSQC spectrum of labelled **1** obtained from (*E*)-(4-^{13}C,4-^{2}H)IPP. The selective incorporation of deuterium can be used to determine the absolute configuration at the investigated methylene position C8. Combining these information with the relative configuration obtained by NOESY results in the absolute configuration for **1** as shown. Black dots represent ^{13}C-labelled carbon atoms.

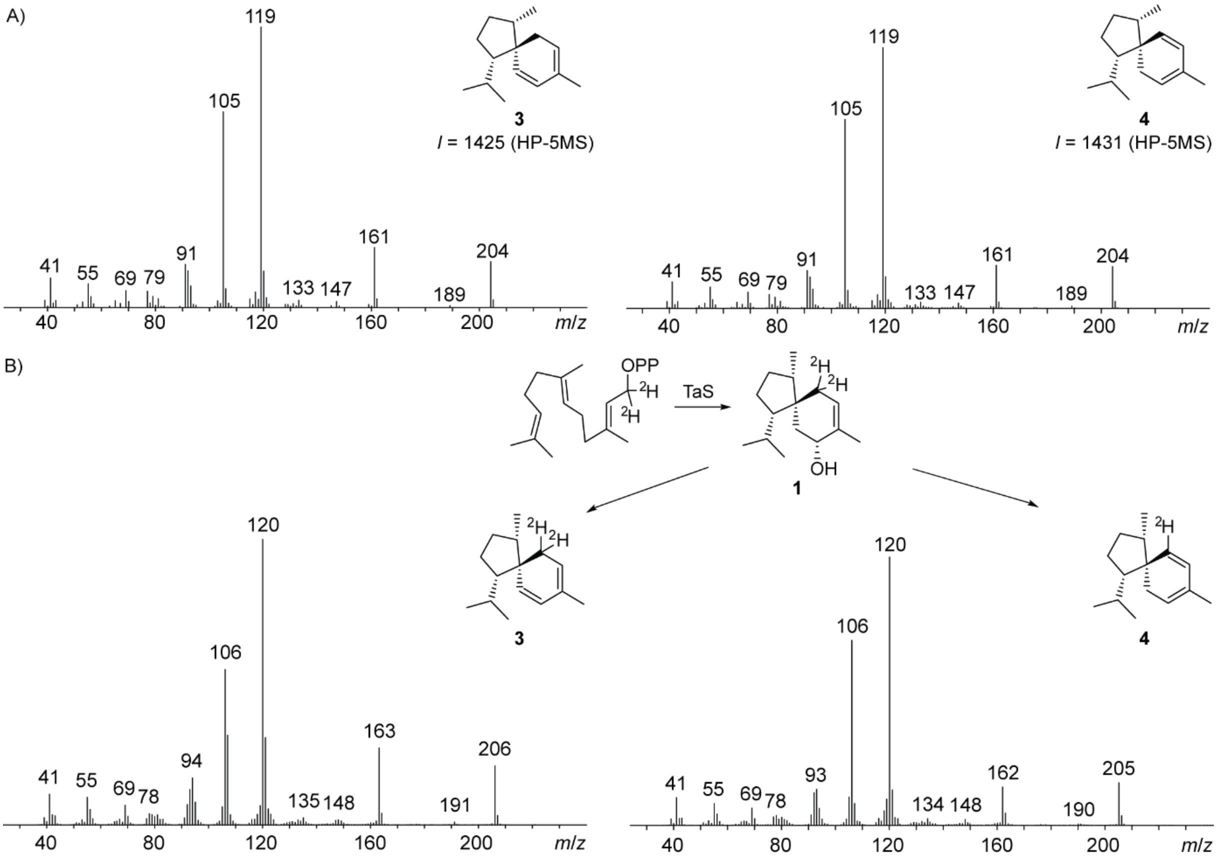

Figure S13. Assignment of acoradienes **3** and **4**. EI-MS spectra of A) unlabelled **3** (left) and **4** (right) and B) labelled **3** (left) and **4** (right) originating from the incubation of (1,1-$^{2}H_2$)FPP with TaS.

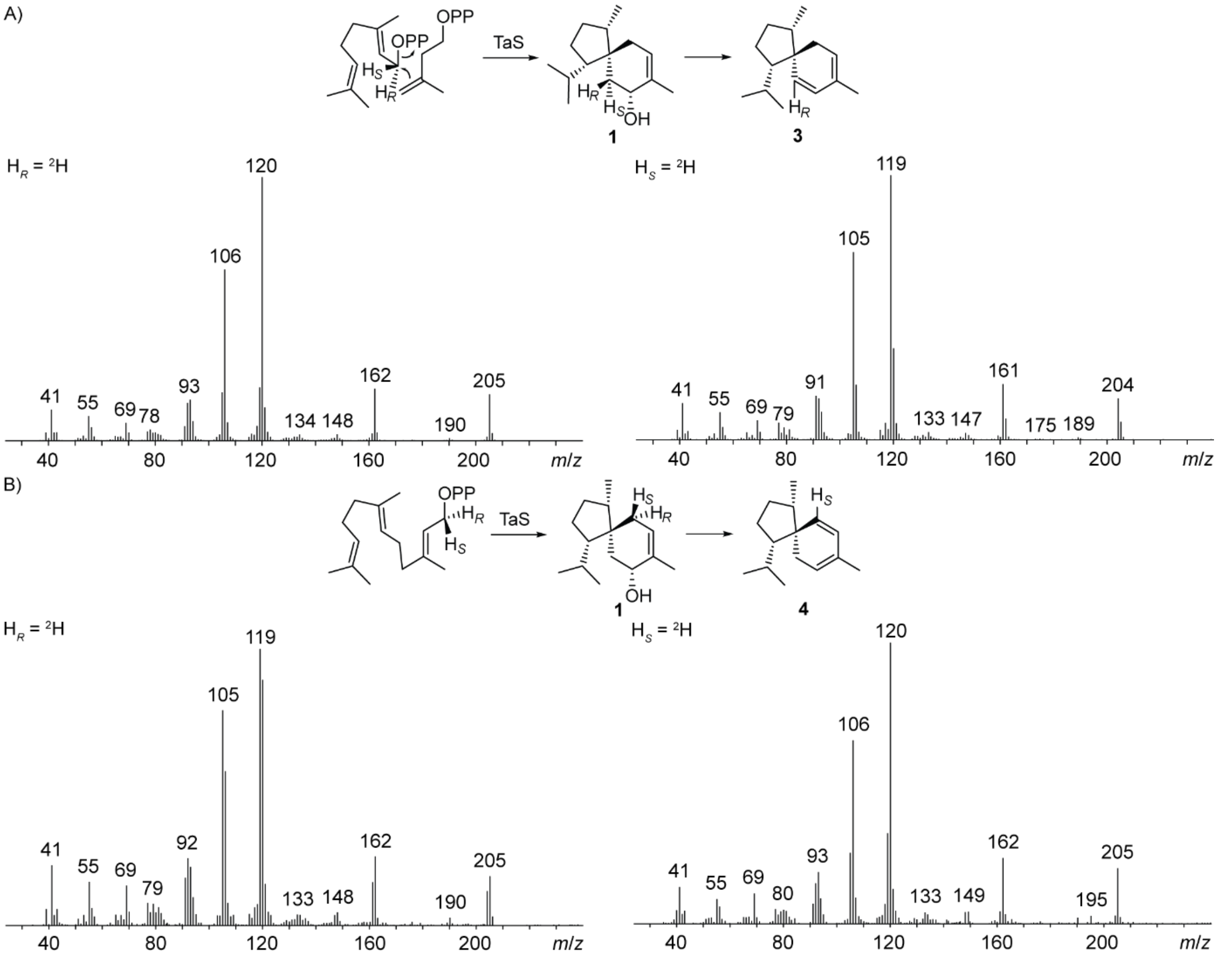

Figure S14. Stereochemical course of the thermal elimination step towards **3** and **4**. EI-MS spectra of A) **3** originating from the conversion of (*R*)-(1-^{2}H)GPP (left) or (*S*)-(1-^{2}H)GPP (right) and IPP with TaS and FPPS and B) **4** originating from the conversion of (*R*)-(1-^{2}H)FPP (left) or (*S*)-(1-^{2}H)FPP (right) and IPP with TaS.

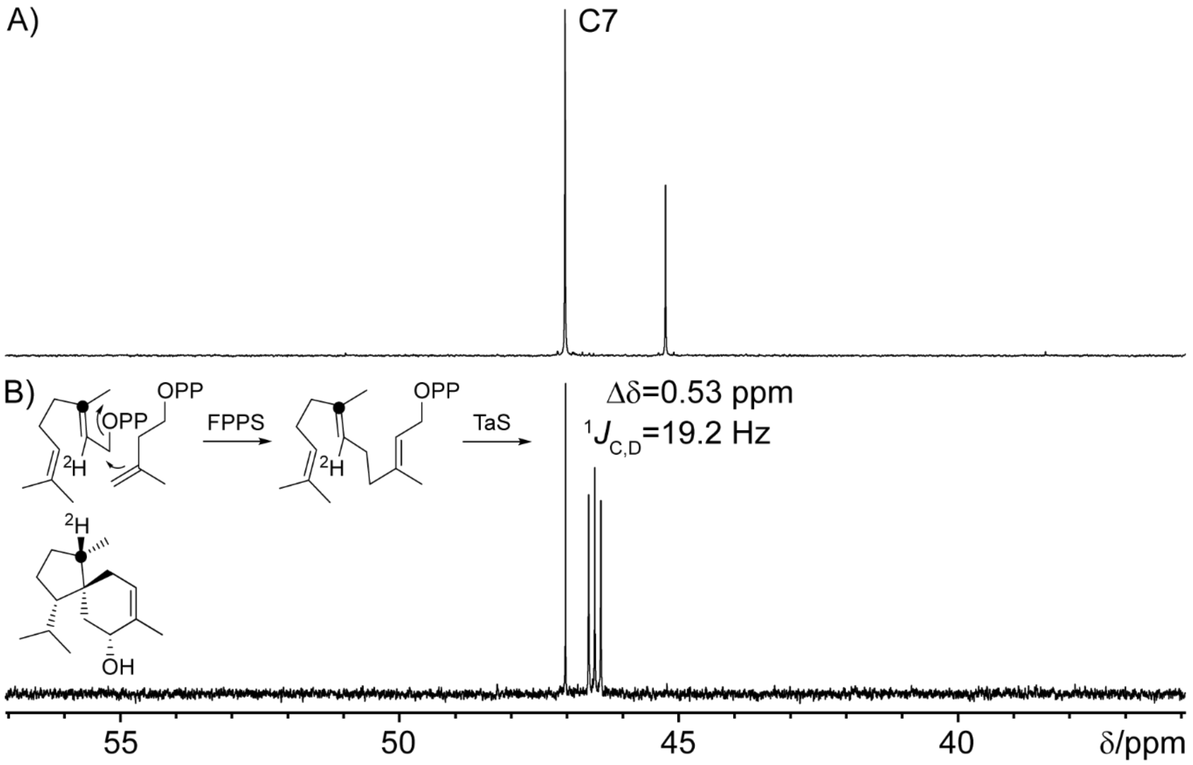

Figure S15. Partial ^{13}C-NMR spectra of A) unlabelled *ent*-**1** and B) an incubation of (3-^{13}C,2-^{2}H)GPP and IPP with TaS and FPPS. The singlet in B) originates from incomplete deuteration in the starting material.

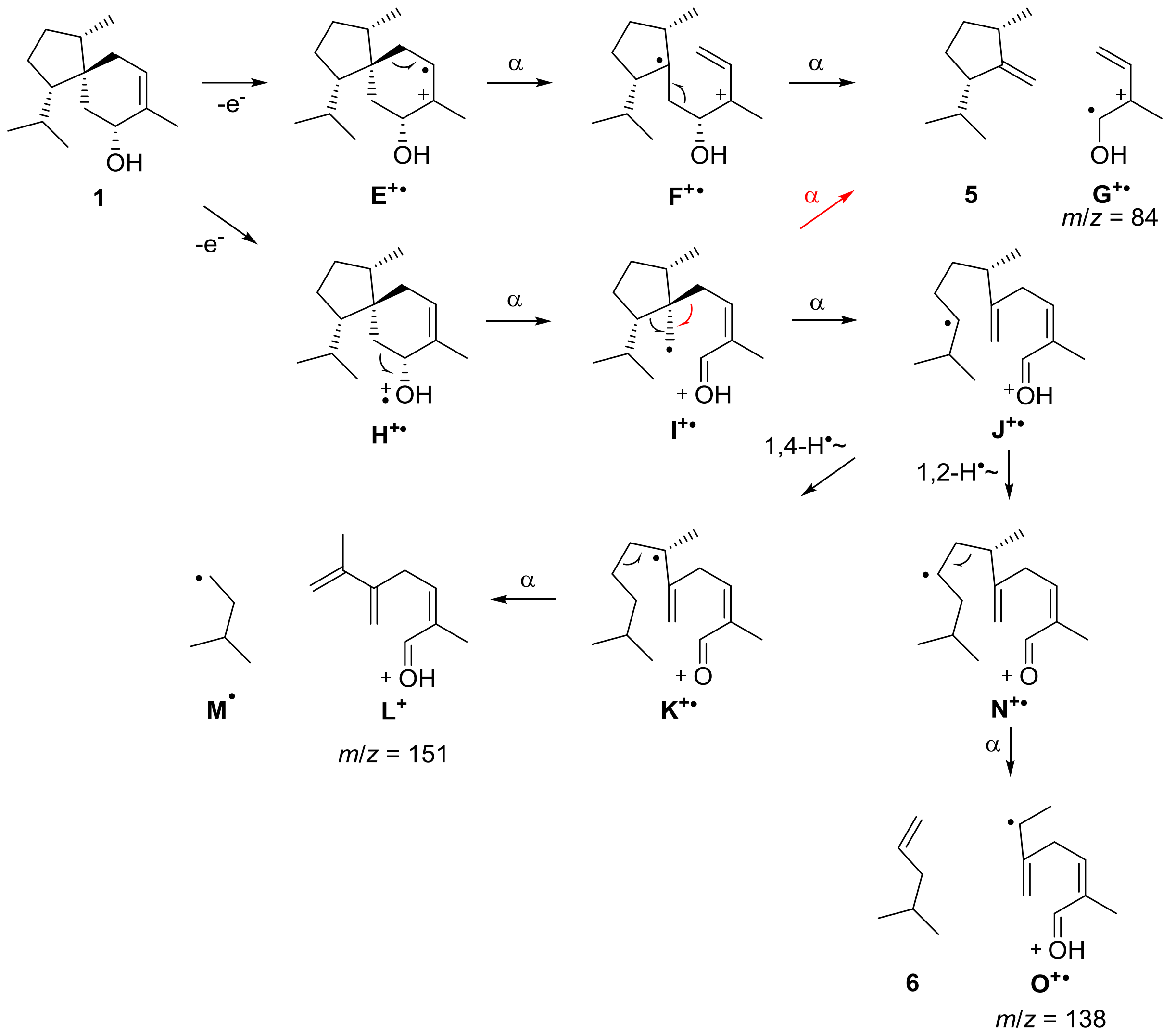

Scheme S1. Originally proposed EI-MS fragmentation mechanism[18] towards the diagnostic fragments *m*/*z* = 151, 138 and 84. A branched pathway is coloured in red.

Ionisation at the olefinic double bond yields radical cation **E**$^{+\cdot}$, which undergoes α-cleavage to **F**$^{+\cdot}$. Another radical cleavage yields **5** and "retro Diels-Alder" fragment **G**$^{+\cdot}$ with *m*/*z* = 84. Starting from an ionisation of one oxygen lone pair with **H**$^{+\cdot}$, two bond breaking events via **I**$^{+\cdot}$ leads to **J**$^{+\cdot}$, which either undergoes a 1,4-hydrogen movement to **K**$^{+\cdot}$ building up **L**$^{+}$ with *m*/*z* = 151 and **M**$^{\cdot}$ by α-fragmentation, or a 1,2-hydrogen shift may lead to **N**$^{+\cdot}$, which gives rise to **O**$^{+\cdot}$ with *m*/*z* = 138 by neutral loss of **6**.

Although the carbon skeletons of **L**$^{+\cdot}$ and **O**$^{+\cdot}$ resembles the results obtained in this study (Figures S17 and 5), the observed hydrogen movements (Figures S18 and 4) disagree with the formation mechanism of **L**$^{+\cdot}$ and **O**$^{+\cdot}$.

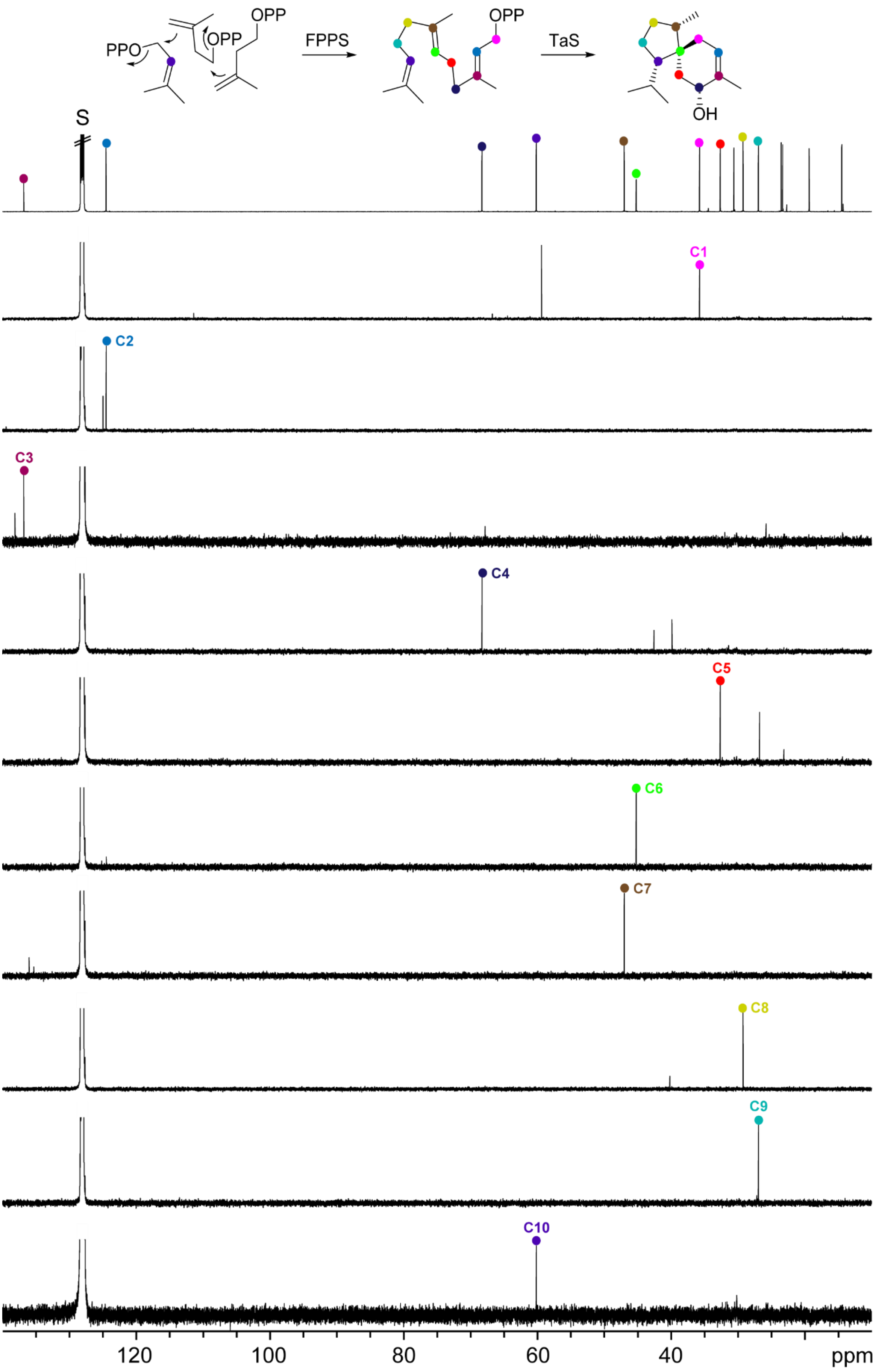

Figure S16. ^{13}C-NMR spectra of unlabelled *ent*-**1** (top) and single-^{13}C labelling experiments for C1 – C15 of FPP. Coloured dots represent the corresponding carbon positions.

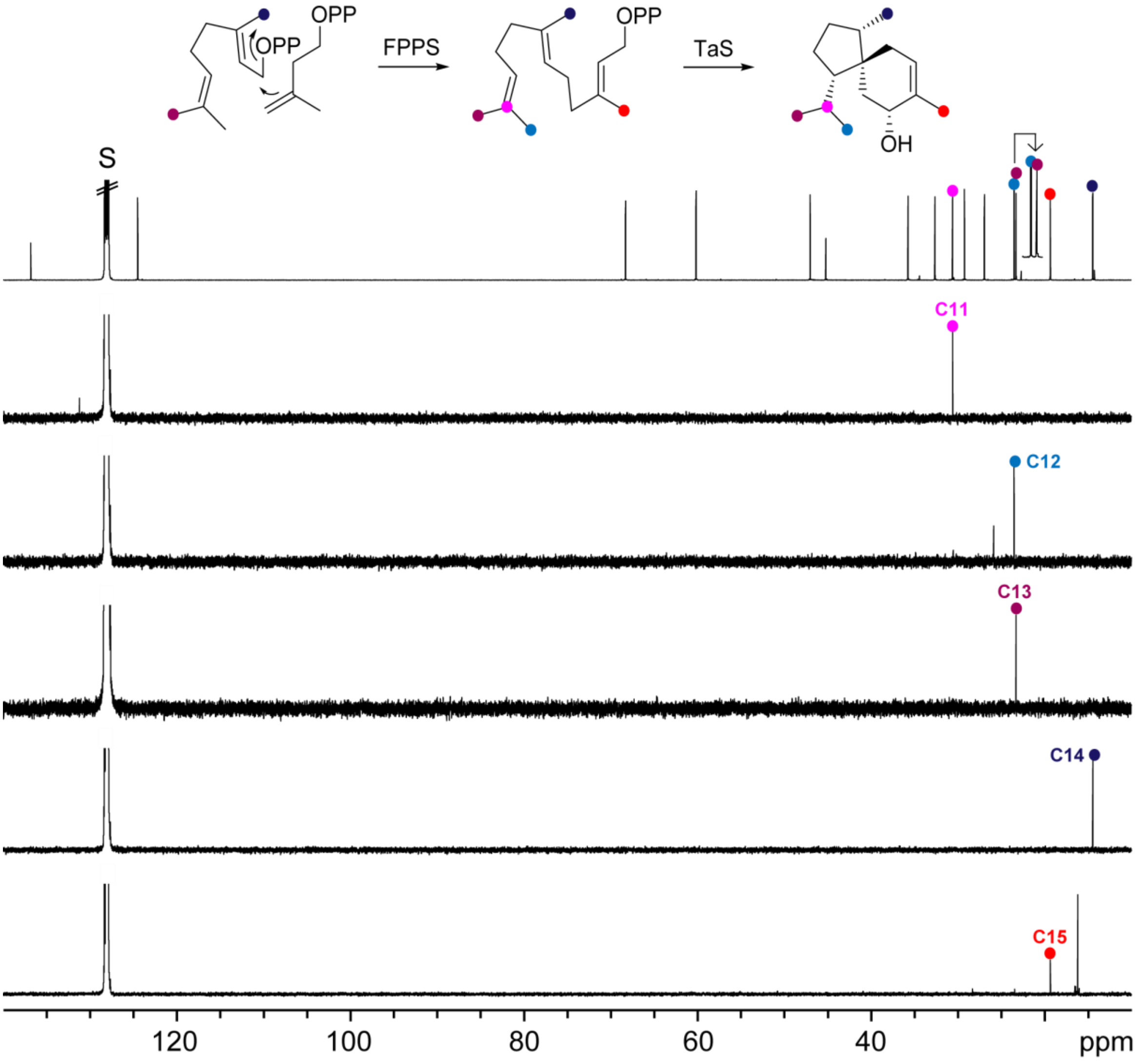

Figure S16 (continued). ^{13}C-NMR spectra of unlabelled *ent*-**1** (top) and single-^{13}C labelling experiments for C1 – C15 of FPP. Coloured dots represent the corresponding carbon positions.

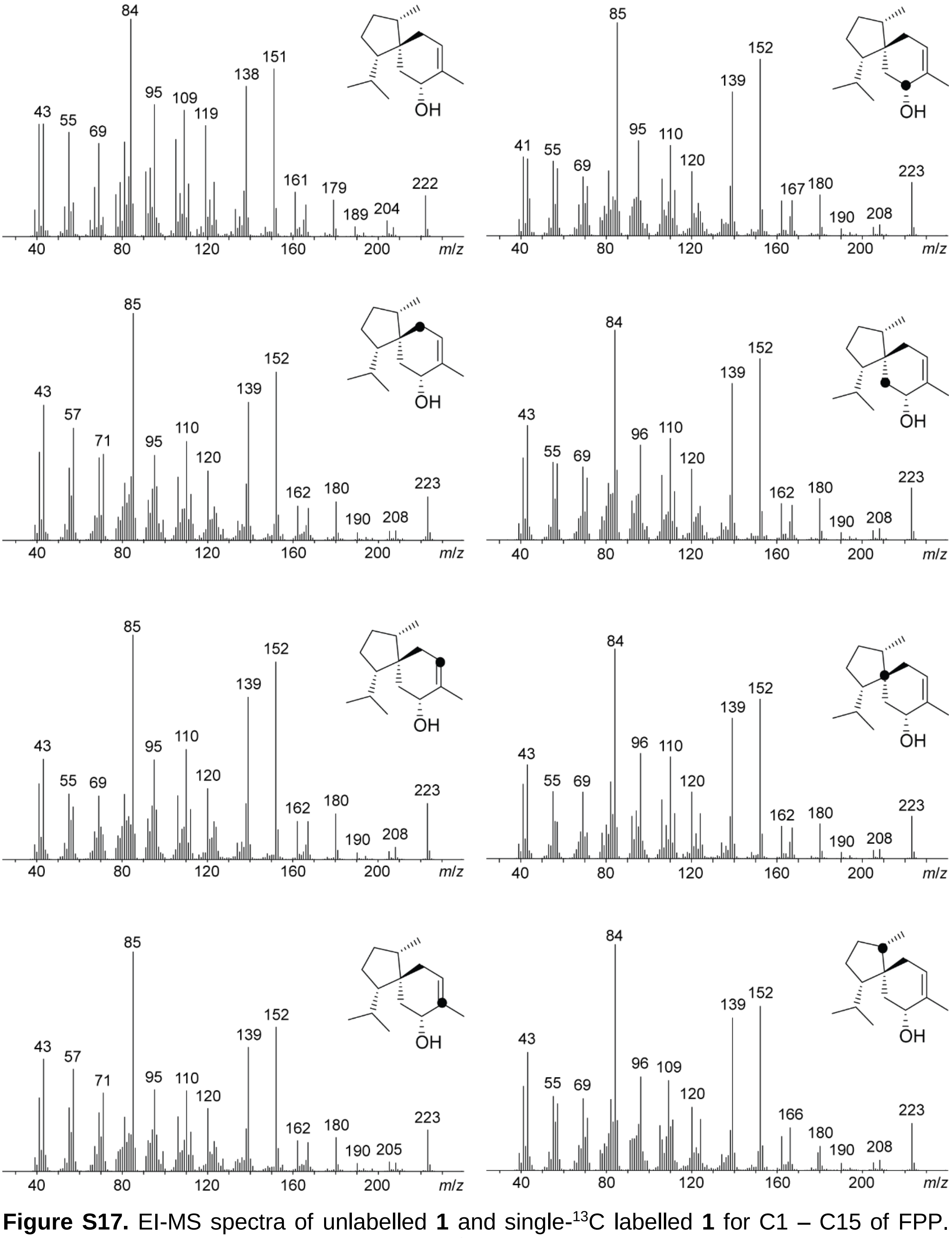

Figure S17. EI-MS spectra of unlabelled **1** and single-^{13}C labelled **1** for C1 – C15 of FPP. Black dots correspond to the position of the ^{13}C-labelled carbon atom.

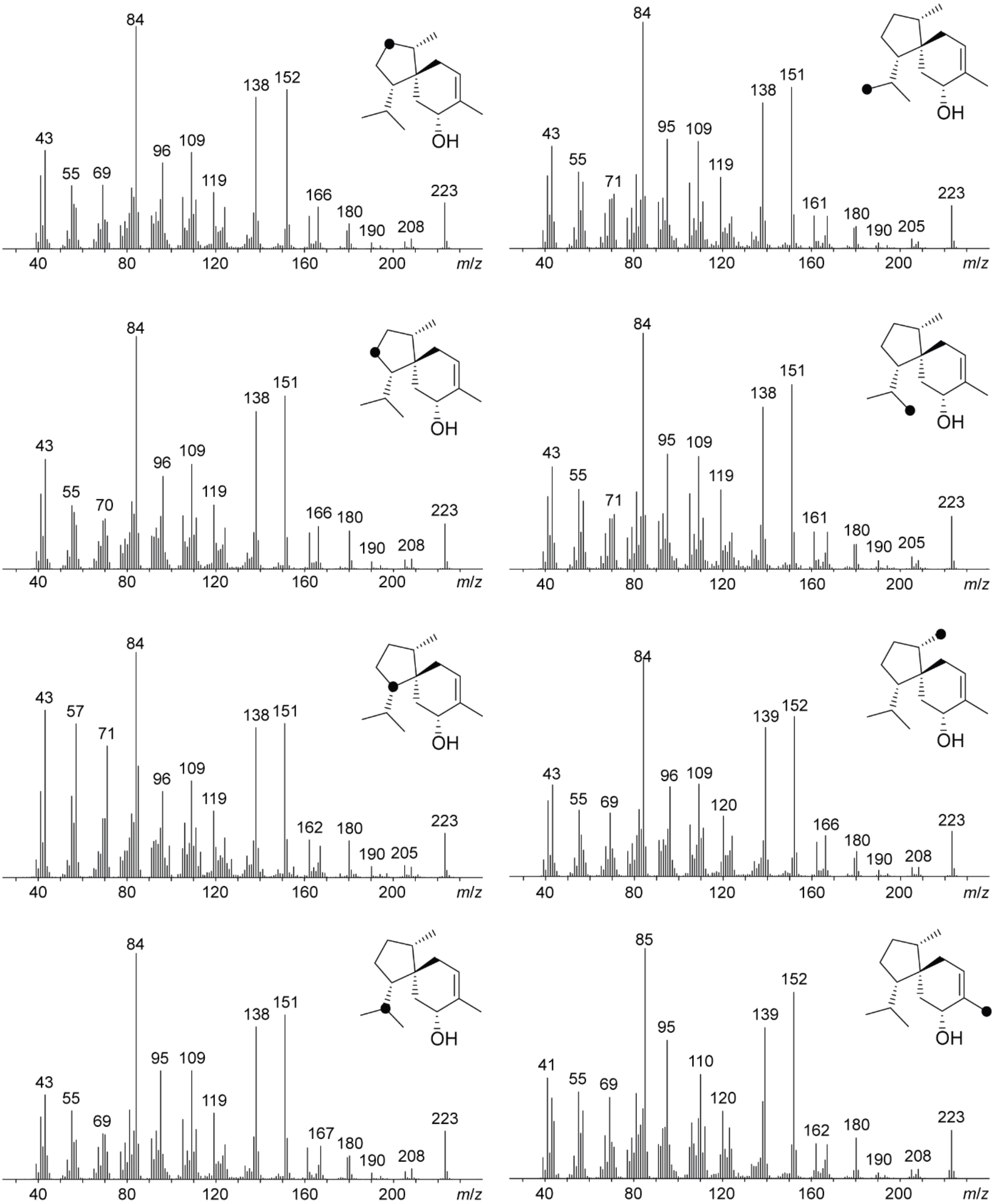

Figure S17 (continued). EI-MS spectra of unlabelled **1** and single-^{13}C labelled **1** for C1 – C15 of FPP. Black dots correspond to the position of the ^{13}C-labelled carbon atom.

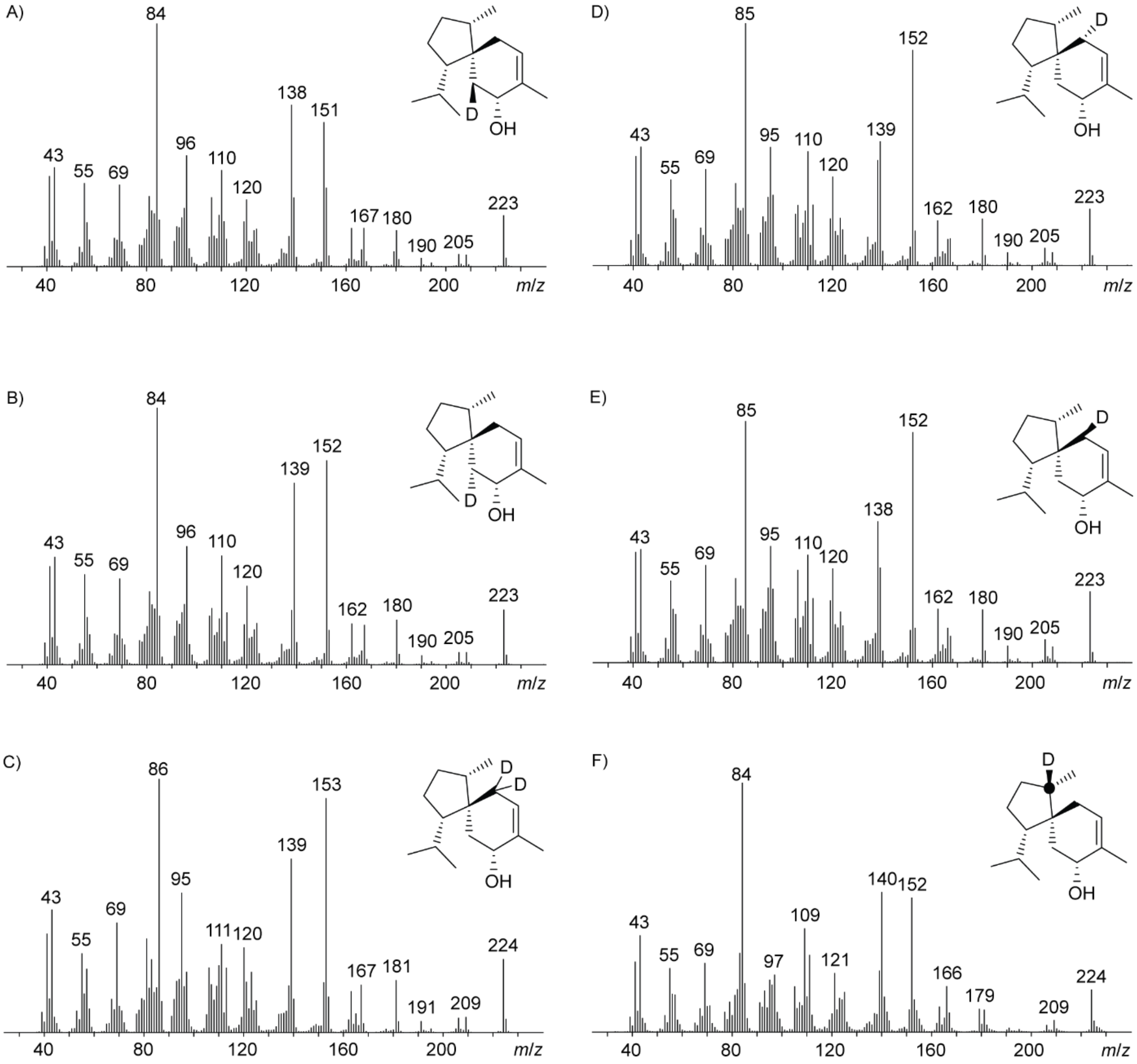

Figure S18. EI-MS spectra of various deuterated isotopomers of **1** to deduce hydrogen movements with respect to the diagnostic fragments originating from incubation experiments using TaS and A) (*R*)-(1-^{2}H)GPP, IPP and FPPS, B) (*S*)-(1-^{2}H)GPP, IPP and FPPS, C) (1,1-$^{2}H_2$)FPP, D) (*R*)-(1-^{2}H)FPP, E) (*S*)-(1-^{2}H)FPP and F) (3-^{13}C,2-^{2}H)GPP, IPP and FPPS. Black dots represent ^{13}C-labelled carbon atoms.

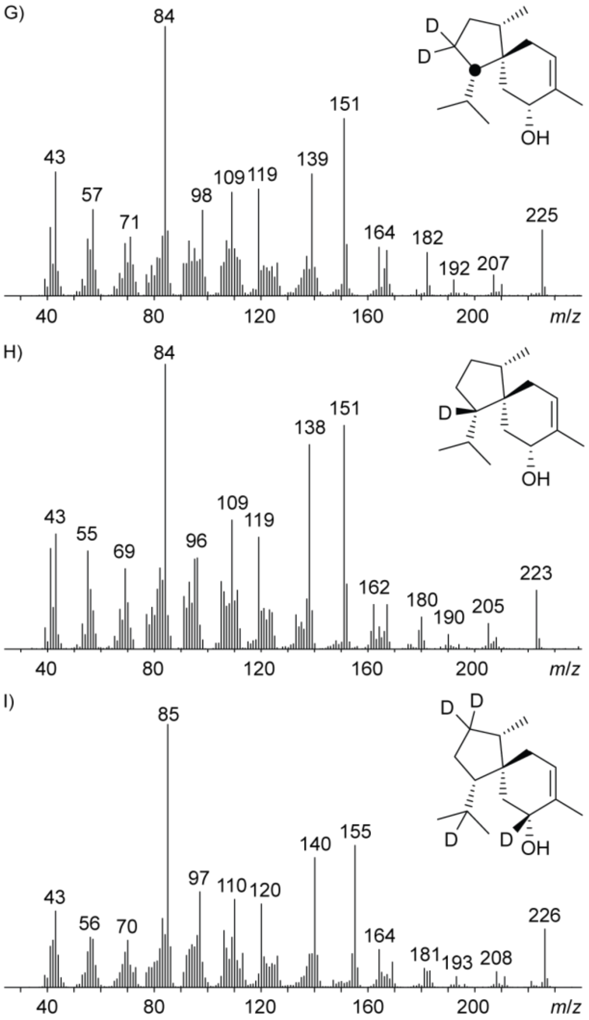

Figure S18 (continued). EI-MS spectra of various deuterated isotopomers of **1** to deduce hydrogen movements with respect to the diagnostic fragments originating from incubation experiments using TaS, FPPS and G) (2-^{13}C,1,1-$^{2}H_2$)DMAPP and IPP, H) (2-^{2}H)DMAPP and IPP and I) DMAPP and (4,4-$^{2}H_2$)IPP. Black dots represent ^{13}C-labelled carbon atoms.

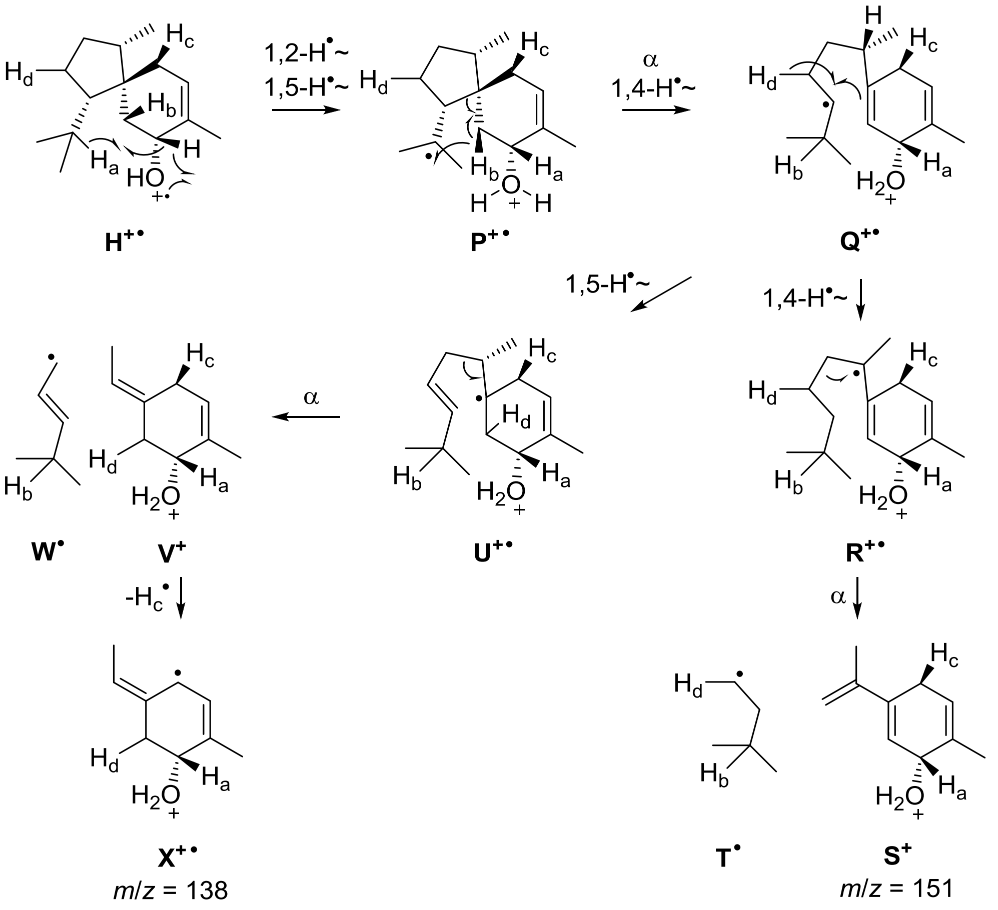

Scheme S2. Proposed EI-MS fragmentation mechanism towards the diagnostic fragments *m*/*z* = 151 and 138 explaining the observed hydrogen movements.

Starting from **H**$^{+\cdot}$, 1,2- and 1,5-hydrogen radical movements (H_a to C4) yields **P**$^{+\cdot}$. Then H_b may shift to C11, accompanied by further α-fragmentation to **Q**$^{+\cdot}$. A 1,4-hydrogen movement results in **R**$^{+\cdot}$, which forms **S**$^{+}$ with *m*/*z* = 151 under loss of **T**$^{\cdot}$. Alternatively, **Q**$^{+\cdot}$ may undergo a 1,5-hydrogen shift of H_d yielding **U**$^{+\cdot}$, which cleaves off **W**$^{\cdot}$. **V**$^{+}$ loses an additional H_c radical to form **X**$^{+\cdot}$ with *m*/*z* = 138.

References

[1] B. Neumann, A. Pospiech, H. U. Schairer, *Trends Genet.,* **1992**, *8*, 332.

[2] J. S. Dickschat, K. A. K. Pahirulzaman, P. Rabe, T. A. Klapschinski, *ChemBioChem* **2014**, *15*, 810.

[3] R. D. Giets, R. H. Schiestl, *Nat. Protoc*. **2007**, *2*, 31.

[4] M. M. Bradford, *Anal. Biochem.* **1976**, *72*, 248.

[5] G. R. Fulmer, A. J. M. Miller, N. H. Sherden, H. E. Gottlieb, A. Nudelman, B. M. Stoltz, J. E. Bercaw, K. I. Goldberg, *Organometallics* **2010**, *29*, 2176.

[6] N. L. Brock, J. S. Dickschat, *Eur. J. Org. Chem.* **2011**, 5167.

[7] J. Rinkel, J. S. Dickschat, *Org. Lett.* **2019**, *21*, 2426.

[8] P. Rabe, J. Rinkel, B. Nubbemeyer, T. G. Köllner, F. Chen, J. S. Dickschat, *Angew. Chem. Int. Ed.* **2016**, *55*, 15420.

[9] L. Lauterbach, J. Rinkel, J. S. Dickschat, *Angew. Chem. Int. Ed.* **2018**, *57*, 8280.

[10] J. Rinkel, P. Rabe, P. Garbeva, J. S. Dickschat, *Angew. Chem. Int. Ed.* **2016**, *55*, 13593.

[11] J. Rinkel, P. Rabe, L. zur Horst, J. S. Dickschat, *Beilstein J. Org. Chem.* **2016**, *12*, 2317.

[12] J. Rinkel, L. Lauterbach, P. Rabe, J. S. Dickschat, *Angew. Chem. Int. Ed.* **2018**, *57*, 3238.

[13] T. Mitsuhashi, J. Rinkel, M. Okada, I. Abe, J. S. Dickschat, *Chem. Eur. J.* **2017**, *23*, 10053.

[14] P. Rabe, L. Barra, J. Rinkel, R. Riclea, C. A. Citron, T. A. Klapschinski, A. Janusko, J. S. Dickschat, *Angew. Chem. Int. Ed.* **2015**, *54*, 13448.

[15] J. Rinkel, J. S. Dickschat, *Beilstein J. Org. Chem.* **2019**, *15*, 1008.

[16] G. Bian, J. Rinkel, Z. Wang, L. Lauterbach, A. Hou, Y. Yuan, Z. Deng, T. Liu, J. S. Dickschat, *Angew. Chem. Int. Ed.* **2018**, *57*, 15887.

[17] J. Rinkel, P. Rabe, X. Chen, T. G. Köllner, F. Chen, J. S. Dickschat, *Chem. Eur. J.* **2017**, *23*, 10501.

[18] C. A. Citron, R. Riclea, N. L. Brock, J. S. Dickschat, *RSC Adv.* **2011**, *1*, 290.
